# MiniCAFE, a CRISPR/Cas9-based compact and potent transcriptional activator, elicits gene expression *in vivo*

**DOI:** 10.1093/nar/gkab174

**Published:** 2021-03-22

**Authors:** Xin Zhang, Sihan Lv, Zhenhuan Luo, Yongfei Hu, Xin Peng, Jie Lv, Shanshan Zhao, Jianqi Feng, Guanjie Huang, Qin-Li Wan, Jun Liu, Hongxin Huang, Bing Luan, Dong Wang, Xiaoyang Zhao, Ying Lin, Qinghua Zhou, Zhen-Ning Zhang, Zhili Rong

**Affiliations:** Cancer Research Institute, School of Basic Medical Sciences, Southern Medical University, Guangzhou 510515, China; Department of Endocrinology, Shanghai Tenth People's Hospital, School of Medicine, Tongji University, Shanghai 200072, China; Zhuhai Institute of Translational Medicine, Zhuhai People's Hospital Affiliated with Jinan University, Jinan University, Zhuhai 519000, China; The Biomedical Translational Research Institute, Faculty of Medical Science, Jinan University, Guangzhou 510632, China; Department of Bioinformatics, School of Basic Medical Sciences, Southern Medical University, Guangzhou 510515, China; Dermatology Hospital, Southern Medical University, Guangzhou 510091, China; Cancer Research Institute, School of Basic Medical Sciences, Southern Medical University, Guangzhou 510515, China; Cancer Research Institute, School of Basic Medical Sciences, Southern Medical University, Guangzhou 510515, China; Cancer Research Institute, School of Basic Medical Sciences, Southern Medical University, Guangzhou 510515, China; Cancer Research Institute, School of Basic Medical Sciences, Southern Medical University, Guangzhou 510515, China; Cancer Research Institute, School of Basic Medical Sciences, Southern Medical University, Guangzhou 510515, China; Zhuhai Institute of Translational Medicine, Zhuhai People's Hospital Affiliated with Jinan University, Jinan University, Zhuhai 519000, China; The Biomedical Translational Research Institute, Faculty of Medical Science, Jinan University, Guangzhou 510632, China; Dermatology Hospital, Southern Medical University, Guangzhou 510091, China; Dermatology Hospital, Southern Medical University, Guangzhou 510091, China; Department of Endocrinology, Shanghai Tenth People's Hospital, School of Medicine, Tongji University, Shanghai 200072, China; Department of Bioinformatics, School of Basic Medical Sciences, Southern Medical University, Guangzhou 510515, China; Dermatology Hospital, Southern Medical University, Guangzhou 510091, China; Department of Development, School of Basic Medical Sciences, Southern Medical University, Guangzhou 510515, China; Cancer Research Institute, School of Basic Medical Sciences, Southern Medical University, Guangzhou 510515, China; Zhuhai Institute of Translational Medicine, Zhuhai People's Hospital Affiliated with Jinan University, Jinan University, Zhuhai 519000, China; The Biomedical Translational Research Institute, Faculty of Medical Science, Jinan University, Guangzhou 510632, China; Translational Medical Center for Stem Cell Therapy & Institute for Regenerative Medicine, Shanghai East Hospital, School of Life Sciences and Technology, Tongji University, Shanghai 200092, China; Cancer Research Institute, School of Basic Medical Sciences, Southern Medical University, Guangzhou 510515, China; Dermatology Hospital, Southern Medical University, Guangzhou 510091, China; Bioland Laboratory (Guangzhou Regenerative Medicine and Health Guangdong Laboratory), Guangzhou 510005, China

## Abstract

CRISPR-mediated gene activation (CRISPRa) is a promising therapeutic gene editing strategy without inducing DNA double-strand breaks (DSBs). However, *in vivo* implementation of these CRISPRa systems remains a challenge. Here, we report a compact and robust miniCas9 activator (termed miniCAFE) for *in vivo* activation of endogenous target genes. The system relies on recruitment of an engineered minimal nuclease-null Cas9 from *Campylobacter jejuni* and potent transcriptional activators to a target locus by a single guide RNA. It enables robust gene activation in human cells even with a single DNA copy and is able to promote lifespan of *Caenorhabditis elegans* through activation of longevity-regulating genes. As proof-of-concept, delivered within an all-in-one adeno-associated virus (AAV), miniCAFE can activate Fgf21 expression in the liver and regulate energy metabolism in adult mice. Thus, miniCAFE holds great therapeutic potential against human diseases.

## Graphical Abstract

**Graphical Abstract:**
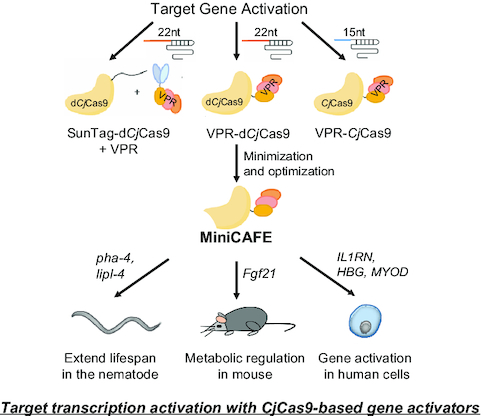
Target transcription activation with CjCas9-based gene activators. Three gene activators, SunTag-dCjCas9/VPR, VPR-dCjCas9, and VPR-CjCas9, are developed. Minimization and optimization of VPR-dCjCas9 leads to creation of MiniCAFE, which is able to activate gene expression and induce corresponding phenotypes in C. elegans, mice and human cells.

## INTRODUCTION

The clustered regularly interspaced short palindromic repeats (CRISPR)/CRISPR-associated protein 9 (Cas9) system, an adaptive immunity system in bacteria and archaea, has been developed into a robust and efficient programmable genome editing tool (1–3). The engineered CRISPR system consists of a Cas9 nuclease and a single guide RNA (sgRNA), which form a complex to recognize the genomic locus of interest via hybridizing of the sgRNA to a 20-nucleotide DNA sequence preceding a defined protospacer-adjacent motif (PAM) and generate a double-strand break (DSB) (4–6). The nuclease-deficient Cas9 mutants, termed dCas9, are able to target and bind genomic DNA without cleavage activity and thus enable RNA-guided transcription activation when coupled to transcription factors or domains (7–9). However, the size of the most widely used *Sp*Cas9 (from *Streptococcus pyogenes*) is about 4.10 kb and exceeds the packaging capacity of some common viral vectors, like recombinant adeno-associated virus (rAAV) , when the promoter and other *cis* elements are included, and even worse when additional transcription factors are included in the transcription activation systems (10,11). Therefore, to fulfill *in vivo* delivery of Cas9-based transcription activators, particularly for clinical disease treatment, it is urgent to identify natural smaller Cas9 orthologs or to develop engineered minimal Cas9 variants.

Several strategies have been reported to address this issue. The most widely used approach is to split the Cas9 transcription activation system (split the Cas9 protein, separate Cas9 from sgRNA, or separate Cas9 from sgRNA and transcription factors in the SAM system) and package in two separate viral vectors (12–15). However, it is a challenge that split-Cas9 proteins are generally expressed poorly and exhibit lower activity, and furthermore from production and regulatory standpoints the all-in-one viral vector is preferred (10,16,17). *Sa*Cas9 (from *Staphylococcus aureus*) is a natural smaller Cas9 ortholog (∼3.16 kb) and has been used to activate transcription *in vitro* and *in vivo* (14,15,18). Engineered *Sp*Cas9 and *Sa*Cas9 with reduced size are able to activate gene expression (18). To minimize the size of a gene activator, VP64 is adopted as a small transcription factor to fuse to Cas9, although the gene activation activity is severely compromised compared to the second generation of activators, like VPR, SAM and SunTag (8,14,15). Currently, *Sa*Cas9 is available and commonly used to activate genes *in vivo* using rAAV, however, the need for more small Cas9 variants with different PAM restriction, low immunogenicity, and high efficacy is still a challenge.

*Cj*Cas9 (from *Campylobacter jejuni*, ∼2.95 kb) is one of the smallest Cas9 orthologs and thus holds promise to expand applicability of CRISPR/Cas9 systems for gene activation *in vivo* (19). Crystal structure of the *Cj*Cas9/sgRNA/DNA complex reveals a unique triple-helix architecture within guide RNA and a distinct contact between *Cj*Cas9 and both strands of target DNA (20). Similar to other Cas9 orthologs, *Cj*Cas9 has been used to introduce indels in cells and to generate knockout mice (19,21). More importantly, delivered via AAV, *Cj*Cas9 can induce indels in mouse muscle cells to treat Duchenne muscular dystrophy (DMD) and in retinal pigment epithelium (RPE) cells to suppress pathological choroidal neovascularization (19,22,23). Additionally, pancreatic cancer modeling can be achieved by *in vivo* multiplex gene editing with *Cj*Cas9 (24). Therefore, *Cj*Cas9 has been well adopted for efficient genome editing *in vivo*. However, *Cj*Cas9-based gene activation system has not been reported.

In the current study, based on *Cj*Cas9, we developed a small and potent transcription activator, miniCAFE, which was able to activate various genes in *C. elegans*, mice and human cells and induced corresponding phenotypes. Thus, miniCAFE can be a universal tool to activate transcription in a wide spectrum of organisms and holds promise for human disease treatment in the future.

## MATERIALS AND METHODS

### Plasmid construction

The plasmids pRGEN-CMV-CjCas9 (#89752), pHRdSV40-NLS-dCas9–24xGCN4_v4-NLS-P2A-BFP-dWPRE (#60910), pHRdSV40-scFv-GCN4-sfGFP-VP64-GB1-NLS (#60904), MS2-P65-HSF1_GFP (#61423), pU6-Cj-sgRNA (#89753) and pU6::*unc-119* sgRNA (#46169) were purchased from Addgene. All sgRNAs targeting the genes of interest were designed through https://benchling.com/ or http://crispor.tefor.net/ and ligated to the corresponding sgRNA expression plasmid. A detailed description of the construction of *Cj*Cas9 fusion proteins is provided in the Supplementary Methods. All sgRNAs, linkers, NLS sequences and tRNA sequences are listed in Supplementary Table S1. All constructs were verified through Sanger sequencing.

### Cell culture

HEK293T, B16, and U2OS cells were maintained in Dulbecco's modified Eagle's medium (DMEM, Life Technologies), and MCF7 were cultured in RPMI 1640 medium (Life Technologies), and all the cells were maintained at 37°C with 5% CO2. All growth media were supplemented with 2 mM l-glutamine (Life Technologies), 100 U/ml penicillin, 100 μg/ml streptomycin (Life Technologies), and 10% FBS. The MCF7 pEF1A-V-D/miniCAFE cell lines were obtained by transfecting corresponding plasmids and selecting positive clones with puromycin. For transient transfection experiments, cells were seeded in 24-well plates, and one day later the cells were transfected with total 500 ng plasmids by polyethylenimine (PEI).

### Mice

C57BL/6 mice were purchased from Slack Laboratory Animal Co., Ltd. (SLAC, Shanghai, China). Male mice aged 8–16 weeks were housed in ventilated cages in a temperature-controlled facility with a 12 h light/12 h dark cycle (lights on 6:00–18:00) and free access to food and water. All mice experiments in this study were performed following the guidelines established by the Animal Experiment Committee of Tongji University and in accordance with the guidelines of School of Medicine, Tongji University.

For insulin tolerance test (ITT) experiments, mice were fasted for 3 h (from 9:00 to 12:00) and injected intraperitoneally with insulin (0.5 U/kg). Blood was collected from the tail vein, and glucose levels were measured with a OneTouch Ultra Glucometer (Johnson & Johnson).

AAV2/8 (AAV2 inverted terminal repeat (ITR) vectors pseudo-typed with AAV8 capsid) viral particles were generated by Brainvta (Wuhan, China). For two-viral-particle injection experiments, rAAV-CMV-V-D-WPRE:rAAV-gFGF21 /rAAV-gGFP (1.25 × 10^10^ vg:10^11^ vg) were delivered to 8-week-old male C57BL/6 mice by tail vein injection. Mice were injected with AAV on day 0 and sacrificed on day 90. The metabolic parameters of mice were measured 79 days after injection. For all-in-one virus injection experiments, 2.5 × 10^11^vg rAAV-gFgf21-CMV-miniCAFE or rAAV-gGFP-CMV-miniCAFE were delivered to 8-week-old male C57BL/6 mice by tail vein injection. Mice were injected with AAV on day 0 and sacrificed on day 36. The metabolic parameters of mice were measured 14 days after injection.

### Transgenic *C. elegans* strains

The wild-type N2 (Bristol) obtained from the Caenorhabditis Genetic Center (CGC) (University of Minnesota, USA) was used as the reference background strain. The miniCAFE transgenic animals were generated by microinjecting the relevant plasmids into the germline of the young adult hermaphrodite worms using a previously described method (25). Injected DNA mixes contained P*dpy-30*::miniCAFE (50 ng/μl) and P*myo-2*::GFP::H2B (5 ng/μl), the additional pharyngeal fluorescence-bearing plasmid was used as co-injection marker. Three independent transgenic strains from microinjection were obtained, which carried miniCAFE expression plasmid stably as an additional chromosome array. Those worms were then microinjected with the sgRNA expression vector containing the gene-specific gRNA sequences targeting the promoter regions, meanwhile, the additional body-wall fluorescence-bearing plasmid P*myo-3*::mCherry (3 ng/μl) was used as co-injection marker. The transgenic progeny of P0 or F1 with fluorescence was picked up for subsequent experiments.

### T7E1 analyses

The purified amplicons were denatured at 95°C for 5 min and annealed in NEB Buffer 2 with a slow ramp down to 4°C, then incubated with T7 endonuclease I (NEB) for 3 h at 37°C and subjected to 2% agarose gel electrophoresis. The primers for T7E1 were listed in Supplementary Table S1.

### Polyacrylamide gel electrophoresis (PAGE) assay

Similar to T7E1 assay, PAGE assay could be used to check the nuclease activities of CRISPR/Cas9 systems (26). Briefly, genomic DNA was isolated using sarkosyl lysis buffer (10 mM Tris pH7.6, 0.5% Sarkosyl, 10 mM NaCl, 10 mM EDTA, 0.1 mg/ml proteinase K) and the target sites were amplified by PCR. The purified amplicons were reannealed to form heteroduplexs, and then subjected to 5% polyacrylamide gel electrophoresis. The primers were listed in Supplementary Table S1.

### Deep-seq

Deep-seq was used to determine the indel frequency. Briefly, on-target sites were amplified by PCR with indexed forward and indexed reverse primers. After purification and concentration normalization, PCR products were pooled into different libraries. Completed libraries were generated by a second round of PCR using the pooled libraries as templates and sequenced with 150-bp paired-end reads on Illumina HiSeq instrument. Pooled samples were demultiplexed according to the indexes within forward and reverse primers for the first round PCR. The primers were trimmed from the raw reads, and clean reads were mapped to the gene sequences with software BWA v0.7.17 (27). SAMtools v0.1.18 was used to obtain sorted bam files (28). Finally, R package Genomic Alignments (29) were used to count Indels in alignment results, and all figures were ploted using R package ggplot2. The primers were listed in Supplementary Table S1.

### Quantitative real-time PCR

Total RNA was extracted from mouse tissue or cells using TRIzol reagent (Invitrogen, Carlsbad, CA, USA) and reverse transcripted using FastQuant RT kit (Tiangen, Shanghai, China). Total RNA was isolated from approximately 500 young adult worms per strain using AG RNAex PRO reagent (Accurate Biology, Changsha, China) by the phenol–chloroform extraction method and reverse transcribed using the PrimeScript™RT reagent Kit with gDNA Eraser (Takara, Dalian, China). Real-time PCR was carried out using SuperReal SYBR Green kit (Tiangen, Shanghai, China) and Lightcycler 96 (Roche, Penzberg, Germany). The primer sequences were listed in Supplementary Table S1.

### RNA-seq

The RNA-seq analysis has been described in our previous work (30). Briefly, total RNA was isolate with TRIzol reagent (Invitrogen, Carlsbad, CA, USA) and mRNA was enriched and fragmented. Libraries were constructed by the following steps: the first strand synthesis, the second strand synthesis, adaptor adding, and PCR amplification. After verification and quantification, libraries were sequenced by HiSeq instrument with 150 bp paired-end module. Hisat2 v2.0.52 was used to build the index of the reference genome and align the paired-end clean reads with the reference genome (31). Then, StringTie v2.23 was used to count the read numbers mapped to each gene (32). Fragments Per Kilobase per Million (FPKM) of each gene was calculated based on the length of the gene and reads count mapped to this gene. Differential expression was defined by a Benjamini-Hochberg adjusted *P* value (*q* value | FDR) of <0.05 and fold change of >2 or <0.5. All figures were plotted using R package ggplot2.

### FACS analysis

All flow cytometry analyses were performed using FlowJo software (TreeStar, USA). Cells were harvested 48 h post-transfection. The cleavage efficiency of *Cj*Cas9 was determined as the proportion of GFP negative cells within the *Cj*Cas9-transfected cells (mCherry- positive).

### Western blotting

Cells or tissues were lysed in RIPA buffer (Tris–HCl 50 mM, pH 7.4, NaCl 150 mM, sodium deoxycholate 0.25%, NP-40 1%, EDTA 1 mM, PMSF 1 mM, Aprotinin 1 mg/ml, leupeptin 1 mg/ml, pepstain 1 mg/ml) and a total of 20 ug of protein was separated by SDS-PAGE electrophoresis and transferred to PVDF membranes (Amersham International, GE Healthcare). Membranes were incubated with blocking solution (5% milk powder in tris-buffered saline–Tween 20 (TBST) ) for 1 h, then with primary antibody (in blocking solution) overnight at 4°C. After several washes in TBST, membranes were incubated with horseradish peroxidase (HRP)-conjugated secondary antibodies for 1 h at room temperature (RT) in blocking solution. Membranes were incubated with ECL western-blotting substrate (Amersham International, GE Healthcare) and imaged by in a Chemidoc XRS system or ChemiDOC (Bio-Rad Laboratories).

The following antibodies were used in this study: anti-HA-tag antibody (MBL, M180–3) for *Cj*Cas9, β-tubulin mouse antibody (Proteintech, 66240-1-Ig), β-actin antibody (Sigma-Aldrich, A3854), UCP1 antibody (Abcam, ab10983), FGF21 (Abcam, ab171941).

### Immunofluorescence

Cells were washed in PBS and fixed in 4% paraformaldehyde for 15 min. After several washes in TBST, cells were permeabilized with 0.3% Triton-X-100 for 10 min, blocked with PBS containing 2% BSA 20 min, and incubation with primary antibodies overnight at 4°C. Then cells were treated with secondary antibody for 45 min at room temperature and mount with DAPI after three times washes in TBST. The following antibodies were used in this study: anti-HA-tag antibody (MBL, M180–3) for *Cj*Cas9, Alexa Fluor 488 goat anti-mouse (Invitrogen, A-11001), and Alexa Fluor 568 donkey anti-mouse (Invitrogen, A-10037).

### Quantification and visualization of *myo-2*::GFP fluorescence in *C. elegans*

To evaluate the fluorescence intensity of *myo-2*::GFP in miniCAFE transgenic worms in the presence or absence of sgRNA targeting the *myo-2* promoter, at least 30 young adult transgenic worms with fluoresce per strain were picked up in M9 containing NaN_3_ (50 mM) to anesthetized, then mounted on 2% agarose pads and observed under a Nikon Ti2-U fluorescence microscope. The total GFP fluorescence intensity of each strain was analyzed and quantified by ImageJ software as previously reported (33). Briefly, the GFP fluorescence intensity in arbitrary units (a.u.) of each worm was measured by drawing the outline of the pharyngeal region using the ImageJ intensity measuring tool. The data shown are the average pixel intensity in each strain (*n* ≥ 30). *P*-value was calculated by a two-tailed Student's *t-*test using GraphPad Prism.

### *C. elegan* fat storage strain and quantification

Fat storage strain was performed as previously described with slight modifications (34). In brief, the young adult transgenic worms were collected with M9 buffer and washed twice, then the samples were fixed, strained by Oil Red O (ORO), and transferred to agar pads for observed and imaged under a Nikon Ti2-U microscope. The ORO intensity quantification was perform as previously reported (35). The total ORO intensity of each strain was analyzed by drawing outline of the intestinal region by ImageJ intensity measuring tool. The mean intensity in arbitrary units (a.u.) of each strain (*n* ≥ 30) were plotted by GraphPad Prism and *P*-value was calculated by a two-tailed Student's *t*-test. All experiments were repeated for three times.

### *C. elegans* lifespan assay

All lifespan experiments were performed at 20°C by using the standard protocols, as previously described (36). Briefly, for each transgenic worms, approximately 100–120 young adults were transferred to a new NGM 6-cm plate containing 10 μM 5-fluoro-2′-deoxyuridine (FUDR, Sigma), and the dead OP50 was seeded on the plate before transfer. The animals were scored daily and the experiments were repeated twice. Statistical analysis was performed using Kaplan–Meier analysis with log-rank (Mantel–Cox) test through SPSS package and *P*-value <0.05 was considered statistically significant.

### Indirect calorimetry

Oxygen consumption (VO_2_) and carbon dioxide production (VCO_2_) were measured in a subgroup of mice using a Comprehensive Lab Animal Monitoring System (Columbus Instruments, Columbus, OH, USA). In brief, male mice housed individually with free access to food and water were acclimatized to the metabolic cages for 24 h prior to a 48 h period of automated recordings every 15 min. Sample air from individual cages was passed through sensors to determine O_2_ and CO_2_ content by an open-circuit Oxymax.

## RESULTS

### The d*Cj*Cas9-based transcriptional activators induced endogenous gene expression

To construct transcriptional activators, we mutated the two key amino acid residues, D8A and H559A (20), within the nuclease domain of *Cj*Cas9 to generate d*Cj*Cas9 (Supplementary Figure S1A). T7E1 and PAGE assays demonstrated loss of DNase catalytic activity of d*Cj*Cas9 at an endogenous site (AAVS1 site) in HEK293T cells and an exogenous site (EGFP site) in a dEGFP HEK293T reporter cell line (a short half-life EGFP variant knock-in line (37)) (Supplementary Figure S1B, C). Moreover, FACS assay showed that d*Cj*Cas9 failed to disrupt EGFP protein expression in the reporter cells, further demonstrating the inability of d*Cj*Cas9 to induce double-strand DNA breaks (Supplementary Figure S1D).

Next, we tried to fuse different transcription activation factors with d*Cj*Cas9 to activate gene expression. In our previous report, the SunTag-VP64 system and p300 fusion protein could enable *Lb*Cpf1 to activate transcription (30). The SunTag system is a signal amplification tool utilizing a repeating peptide array to recruit multiple copies of a protein fused to the peptide-recognizing antibody, which has been widely used for fluorescence imaging and gene expression (30,38,39). The catalytic histone acetyltransferase (HAT) core domain of the human E1A-associated protein p300 (P300 core domain) could activate gene expression when combined with CRISPR systems (30,40). Similarly, we constructed a d*Cj*Cas9-SunTag-VP64 system by fusing ten copies of the GCN4 peptide repeat to the N- or C-terminus of d*Cj*Cas9 to recruit the transcription factor VP64 to the target site and a d*Cj*Cas9-P300 system by fusing P300 core domain to the N- or C-terminus of d*Cj*Cas9 (Supplementary Figure S1E). Unfortunately, either of the two systems barely activated *MYOD* and *IL1RN* when transiently transfected into HEK293T cells (Supplementary Figure S1F).

**Figure 1.**
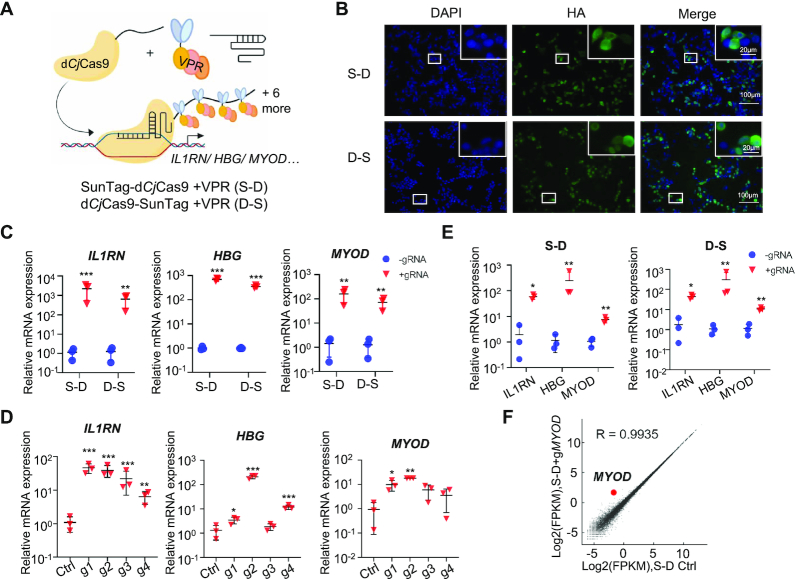
Activation of endogenous genes with a d*Cj*Cas9-SunTag-VPR system. (**A**) Schematic of the d*Cj*Cas9-SunTag-VPR system. 10× SunTag was fused to the N- or C-terminus of a DNase-dead d*Cj*Cas9 (termed S-D and D-S, respectively). By binding to the SunTag, scFv-GCN4-sfGFP-VPR could be recruited to the sgRNA target site and activate transcription. VPR, the truncated tripartite activation domains of VP64, p65 and RTA. (**B**) The subcellular localization of the d*Cj*Cas9-SunTag fusion proteins in HEK293T cells revealed by immunofluorescence staining. An HA tag was fused to the C-terminus of d*Cj*Cas9. (**C**) Targeted gene activation guided by pooled sgRNAs (four sgRNAs for each gene) with the d*Cj*Cas9-SunTag-VPR system. (**D**) Targeted gene activation guided by a single sgRNA with the S–D system. (**E**) Multiplexed gene activation with the d*Cj*Cas9-SunTag-VPR system. (**F**) The gene activation specificity of the S-D system. Gene expression plot generated from RNA-seq data from HEK293T cells co-transfected with S-D and the sgRNA2 targeting *MYOD* compared to that transfected with the corresponding S–D plasmid only. *R* indicates Pearson's correlation coefficient. Average of two biological replicates was shown. For C–E, quantitative RT-PCR revealed relative mRNA expression of *IL1RN*, *HBG*, and *MYOD* in HEK293T cells co-transfected with either S-D or D-S plasmids and four sgRNAs targeting the promoter region of each gene (**C**) or with S-D plasmid and four single sgRNAs targeting the promoter region of each gene (**D**) or with either S–D or D–S plasmids and the pooled three sgRNAs (sgRNA1, sgRNA2 and sgRNA2 for *IL1RN*, *HBG*, and *MYOD*, respectively) (**E**). Mean values are presented with S.D., *n* = 3 independent experiments. For each experiment, fold changes of mRNA expression in tested samples (transfected with the plasmids encoding S–D/D–S and sgRNA) versus that in control samples (transfected with plasmids encoding S–D/D–S and the backbone plasmid for sgRNA, herein termed ‘without sgRNA’) were shown. *P <0.05, **P <0.01, ***P <0.001 (Student's *t*-test for C and E, one-way ANOVA test for D, tested sample versus control sample).

The tripartite activator VP64-p65-Rta has been reported to be more robust to activate gene expression than most known transcriptional activators, including VP64 (8,41). And several truncated VP64-p65-Rta tripartite activators have been developed to minimize the size while maintained equal gene activation ability (42). Thus, we replaced VP64 with a truncated VP64-p65-Rta tripartite activator (referred to as VPR) to generate a d*Cj*Cas9-SunTag-VPR system (Figure 1A). The SunTag-d*Cj*Cas9 fusion protein, within which the ten copies of the GCN4 peptide repeat were fused at the N-terminus of d*Cj*Cas9, was termed as S-D system. And the d*Cj*Cas9-SunTag fusion protein, within which the ten copies of the GCN4 peptide repeat were fused at the C-terminus of d*Cj*Cas9, was termed as D–S system (Figure 1A). Immunofluorescence staining in HEK293T cells showed that the S–D and D–S fusion proteins were detectable in the nucleus (Figure 1B), and Western blotting showed predicted sized protein bands (Supplementary Figure S1G). Quantitative RT-PCR revealed transcriptional activation of *IL1RN*, *HBG* and *MYOD* genes in HEK293T cells when each promoter region was targeted by four sgRNAs (Figure 1C). Using S–D system, a single sgRNA could activate transcription although with various capabilities (Figure 1D). The dCjCas9-SunTag-VPR system could also activate gene expression in other cell lines, including U2OS, a human bone osteosarcoma cell line, and MCF7, a human breast cancer cell line (Supplementary Figure S2). Additionally, the mRNA expression of *IL1RN*, *HBG* and *MYOD* gene could be simultaneously stimulated when each promoter region was targeted by a single sgRNA (sgRNA1, sgRNA2 and sgRNA2 for *IL1RN*, *HBG* and *MYOD*, respectively) (Figure 1E). We noted that S–D system was more potent than D–S system to activate gene expression (Figure 1C and Supplementary Figure S2A, B). And finally, using RNA-seq, we tested the specificity of gene activation with S–D system. Compared with the control group transfected with all the plasmids except the sgRNA, the expression level of untargeted genes in the groups co-transfected with S-D system and sgRNA2 targeting *MYOD* promoter region was not broadly affected, and the expression of *MYOD* was significantly increased (FDR < 0.05) (Figure 1F). In summary, based on the small *Cj*Cas9, we successfully developed the d*Cj*Cas9-SunTag-VPR system as an efficient transcription activation tool with potential high specificity.

**Figure 2.**
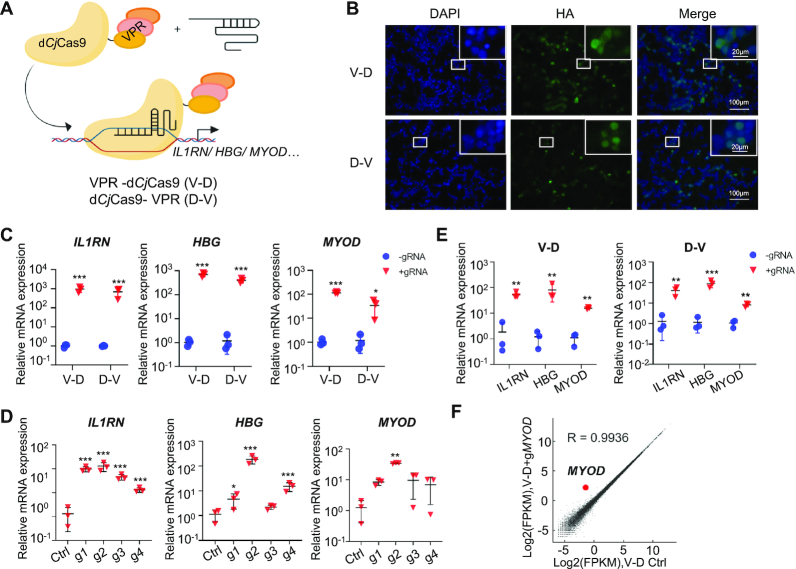
Activation of endogenous genes with a VPR-d*Cj*Cas9 system. (**A**) Schematic of the VPR-d*Cj*Cas9 system. VPR was fused to the N- or C-terminus of d*Cj*Cas9 (termed V–D and D–V, respectively). (**B**) The subcellular localization of the VPR-d*Cj*Cas9 fusion proteins in HEK293T cells revealed by immunofluorescence staining. (**C**) Targeted gene activation guided by pooled sgRNAs with the VPR-d*Cj*Cas9 system. (**D**) Targeted gene activation guided by a single sgRNA with the V-D activator. (**E**) Multiplexed gene activation with the VPR-d*Cj*Cas9 system. (**F**) The gene activation specificity of the V-D activator. Average of two biological replicates was shown. For C–E, mean values are presented with S.D., *n* = 3 independent experiments. The experiments in Figure 2 were similar to that in Figure 1 except using the VPR-d*Cj*Cas9 system instead of the d*Cj*Cas9-SunTag-VPR system. * *P* <0.05, ** *P* <0.01, *** *P* <0.001 (Student's *t*-test for C and E, one-way ANOVA test for D, tested sample versus control sample).

The d*Cj*Cas9-SunTag-VPR system consists of a 10XGCN4-d*Cj*Cas9 fusion protein and an antibody-VPR fusion protein and is a complex and large-sized system. To establish a simple and small-sized tool, we directly fused the VPR to the N- or C-terminus of d*Cj*Cas9 (termed V–D and D–V, respectively) and developed a VPR-d*Cj*Cas9 system (Figure 2A). Similar to the d*Cj*Cas9-SunTag-VPR system, the VPR-d*Cj*Cas9 fusion proteins were detectable in the nucleus, activated gene expression with pooled sgRNAs or a single sgRNA in various cell types, including HEK293T, U2OS, and MCF7 cells, and exhibited high specificity (Figure 2 and Supplementary Figure S3). We noted that the VPR-d*Cj*Cas9 system performed as well as, if not better than, the d*Cj*Cas9-SunTag-VPR system. And similar to the d*Cj*Cas9-SunTag-VPR system, we also noted that V–D fusion protein with VPR at the N-terminus of d*Cj*Cas9 was more potent than D–V fusion protein to activate transcription (Figure 2C and Supplementary Figure S3A, B). Therefore, we focused on V–D in our further experiments.

### Multiplexed orthogonal genome editing and transcriptional activation with a catalytically active *Cj*Cas9 nuclease fused with VPR

**Figure 3.**
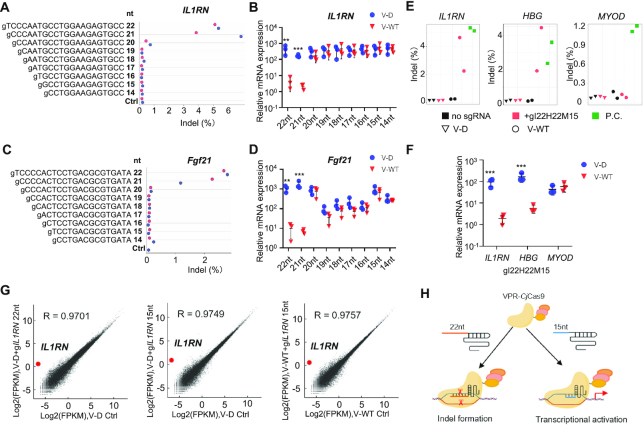
Multiplexed orthogonal genome editing and transcriptional activation with a VPR-*Cj*Cas9 fusion nuclease. (**A**) Genome editing efficiency of the VPR-*Cj*Cas9 fusion nuclease at the *IL1RN* site revealed by Deep-seq. (**B**) Relative mRNA expression of *IL1RN* revealed by qRT-PCR. V-D, VPR-d*Cj*Cas9 fusion protein. V-WT, VPR-*Cj*Cas9 fusion protein. For a & b, human HEK293T cells were co-transfected with either V-D or V-WT plasmids and *ILRN* targeting sgRNAs with indicated length. Half of the cells was used to extract RNA for RT-PCR and half was used to extract DNA for Deep-seq. Ctrl, V-WT transfection only (without sgRNA). (**C**, **D**) Genome editing and gene activation mediated by long and short sgRNAs at the *Fgf21* site in mouse cells. The experiments in C & D were similar to that in A & B except using B16 cells. (E, F) Multiplexed orthogonal genome editing at the *IL1RN* and *HBG* sites and gene activation of *MYOD* in HEK293T cells. Indel formation was revealed by Deep-seq (**E**), and relative mRNA expression was revealed by qRT-PCR (**F**). gI22H22M15, a combination of three sgRNAs including a 22-nt g*IL1RN*, a 22-nt g*HBG*, and a 15-nt g*MYOD*. P.C., positive control, HEK293T cells transfected with WT *Cj*Cas9 and corresponding 22-nt sgRNA plasmids. (**G**) The gene activation specificity of the V-WT activator. Average of two biological replicates was shown. (**H**) Schematic of the VPR-*Cj*Cas9 based orthogonal system. 15-nt sgRNAs activate gene expression, while 22-nt sgRNAs induce gene editing. For B, D and F, mean values are presented with S.D., *n* = three independent experiments. **P <0.01, ***P <0.001 (Student's *t*-test, V–D sample versus corresponding V-WT sample).

Simultaneous orthogonal gene activation and genome editing for multiplex genes with wild type (WT) *Sp*Cas9 or *As*Cas12a (*As*Cpf1) fused to regulation factors has been reported (43–46). However, wide application of this strategy is limited by the large size, which is an obstacle for efficient delivery *in vitro* and *in vivo*. Since *Cj*Cas9 is much smaller than *Sp*Cas9 and *As*Cas12a, we tried to develop a small multiplex genome editor by fusing VPR to the N-terminus of WT *Cj*Cas9 (VPR-WT*Cj*Cas9, V-WT). Deep-seq and T7E1 assays showed that V-WT cleaved genomic DNA at the *IL1RN* promoter region in human HEK293T cells when combined with 21-nt or 22-nt sgRNAs and failed to induce indels when combined with 14- to 20-nt sgRNAs (Figure 3A and Supplementary Figure S4A). V-WT activated *IL1RN* expression to the same extent as V-D when co-transfected with 14- to 20-nt sgRNAs and failed to activate gene expression when co-transfected with 21-nt or 22-nt sgRNAs (Figure 3B). In addition, we observed similar phenomena at another gene site, *MYOD*, except that 20-nt sgRNA induced indels instead of gene activation (Supplementary Figure S4B-D). Next, we tested six sgRNAs targeting *Fgf21* promoter region in mouse B16 cells and used the best sgRNA2 to check whether the V-WT system functioned in mouse cells (Supplementary Figure S4E). Consistently, similar results were observed in mouse B16 cells (Figure 3C, D, and Supplementary Figure S4F).

In the tested *IL1RN*, *MYOD* and *Fgf21* sites, 15-nt and 22-nt sgRNAs exhibited potent gene activation and DNA cleavage ability, respectively. Therefore, we used 15-nt and 22-nt sgRNAs for orthogonal multiplex gene engineering in the following experiments. In a three-gene set, we used a 22-nt sgRNA1 for *IL1RN*, a 22-nt sgRNA2 for *HBG*, and a 15-nt sgRNA2 for *MYOD*. Deep-seq and T7E1 assays showed that V-WT induced indels at the *IL1RN* and *HBG* sites but not the *MYOD* site (Figure 3E and Supplementary Figure S4G). Quantitative RT-PCR assay revealed robust gene activation for *MYOD* but not *IL1RN* or *HBG* (Figure 3F). In another three-gene set, we replaced the 22-nt sgRNA1 for *IL1RN* with a 15-nt sgRNA1 and observed DNA cleavage only at the 22-nt sgRNA targeted *HBG* site and gene activation only at the 15-nt sgRNA targeted *IL1RN* and *MYOD* sites (Supplementary Figure S4H–J). These results demonstrated that the V-WT system could induce multiplexed orthogonal gene editing and activation.

To check whether a truncated 15-nt sgRNA or WT *Cj*Cas9 could affect the specificity of gene activation, we profiled genome-wide gene expression by RNA-seq using *IL1RN* as the target gene in HEK293T cells. Similar to V–D co-transfected with a 22-nt sgRNA1, V–D or V-WT co-transfected with a 15-nt sgRNA1 showed comparable high specificity (Figure 3G). In addition, no broad different expression was observed between the three groups (Supplementary Figure S4K). All the above results demonstrated that VPR-*Cj*Cas9 was able to cleave genomic DNA when combined with a long 22-nt sgRNA and to activate gene expression with high specificity when combined with a short 15-nt sgRNA, and thus realized multiplexed orthogonal genome editing and transcriptional activation (Figure 3H).

### Minimization and optimization of the compact VPR-d*Cj*Cas9 transcriptional activation system

Owing to the large size of Cas proteins, several CRISPR-mediated transcription modification systems have been used in postnatal mammals (13–15,47). AAV vectors have low immunogenicity and broad tissue-specific tropism and thus hold great potential for clinic application (48). However, the major challenge for AAV vectors is the limited carrying capacity, generally less than 5.2 kb (49). Although VPR-d*Cj*Cas9 is small enough to be packaged into an AAV vector, we tried to minimize and optimize the system to improve the payload capacity and transduction efficiency when delivered by AAV and thus broaden its application.

**Figure 4.**
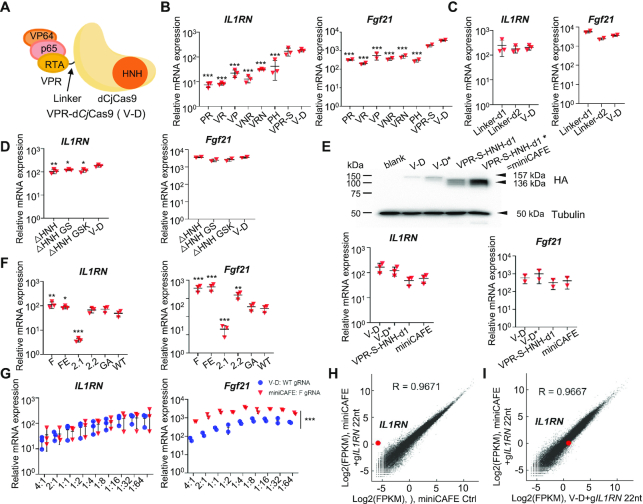
Minimization and optimization of the VPR-d*Cj*Cas9 system. (**A**) Schematic of VPR-d*Cj*Cas9 fusion protein. (**B**) Screening the transcription factors that fused to d*Cj*Cas9. PR, p65-RTA; VR, VP64-RTA; VP, VP64-p65; VNR, VP64-NANOG-RTA; VRN, VP64-RTA-NANOG; PH, p65-HSF1; VPR-S, VPR with a more shortened p65 and shortened linkers between the three transcription factors. (**C**) Shortening the linker between VPR and d*Cj*Cas9. Linker-d1, deleting 33 aa including 2xSV40 NLS; Linker-d2, deleting 11 aa. (**D**) Minimization of d*Cj*Cas9. The HNH domain (495–609 aa) was deleted and the remained N- and C-terminal domains were ligated with no linker, GGGSGG linker, or GSK linker. (**E**) Codon optimization of VPR-d*Cj*Cas9 fusion protein. Upper panel, Western blotting of d*Cj*Cas9 fusion proteins in transfected HEK293T cells. Bottom panel, gene activation with the indicate activators. The fusion proteins with codon-optimized VP64 were indicated with asterisks. VPS-S-HNH-d1* was termed as miniCAFE. (**F**) Optimization of sgRNA scaffold. The structures of these sgRNAs were illuminated in Supplementary Figure S5D. (**G**) Optimization of the molar ratio of the transfected gene activator plasmid to sgRNA plasmid. (**H**) The gene activation specificity of miniCAFE. (**I**) The comparison of gene expression profile between miniCAFE and V-D. For B–G, qRT-PCR revealed relative mRNA expression of *IL1RN* in HEK293T cells and of *Fgf21* in B16 cells. Mean values are presented with S.D., n = 2–3 independent experiments. *P <0.05, **P <0.01, ***P <0.001 (One-way ANOVA test for B–D, tested sample VS V-D sample; Student's *t*-test for E, V–D* versus V–D, and miniCAFE VS VPR-S-HNH-d1; one-way ANOVA test for F, tested sample versus WT sgRNA sample; Student's *t*-test for G, miniCAFE/F gRNA VS corresponding V-D/WT gRNA). For H and I, gene expression plots generated from RNA-seq data from HEK293T cells co-transfected with miniCAFE and 22-nt g*IL1RN*1 with F scaffold. Ctrl, miniCAFE transfected only. Average of two biological replicates was shown.

First, we tried to minimize VPR-d*Cj*Cas9 fusion protein, which consisted of a VP64-p65-Rta tripartite activator, a d*Cj*Cas9, and a linker between the two moieties (Figure 4A). To shorten the VP64-p65-Rta tripartite activator, we tried to delete one factor or to replace the factor with a smaller transcription activation domain, including the transcription activation domains of NANOG and HSF1, which have been reported to be potent gene activators (50,51). However, these fusion proteins induced less potent gene activation for human *IL1RN* and mouse *Fgf21* than V–D (Figure 4B). Next, we replaced p65 with a shorter truncation and shortened the linkers between VP64, p65, and RTA (termed VPR-S), and VPR-S maintained comparable gene activation capability to V–D (Figure 4B). For the linker between VPR and d*Cj*Cas9, we tried two short versions and found that neither of them compromised the activity (Figure 4C). Like *Sp*Cas9 and *Sa*Cas9, *Cj*Cas9 possesses a similar structure, and the HNH and RuvC domains are critical for DNA cleavage but not for DNA binding (20). It has been reported that the mutants with deletion within the HNH and RuvC domains remain comparable activity in *Sp*Cas9- and *Sa*Cas9-based gene activation systems (18). Therefore, we constructed the HNH-truncation (Δ495–609 aa) and RuvC-truncation (Δ243–426 aa) with no linker, GSK linker and GS linker, respectively, and found that HNH-truncations slightly compromised the gene activation capability for *IL1RN* activation in human cells and maintained comparable gene activation capability for *Fgf21* activation in mouse cells, while RuvC-truncations lost gene activation ability (Figure 4D and data not shown). By all the above approaches, we minimized the size of VPR-d*Cj*Cas9 from 4.1 to 3.6 kb.

Next, we tried to optimize the VPR-d*Cj*Cas9 fusion protein to increase gene activation efficiency. We noted that V–D and D–V showed much lower protein expression level than *Cj*Cas9, d*Cj*Cas9, S–D and D–S (Supplementary Figure S1G), indicating the VPR moiety might decrease VPR-d*Cj*Cas9 expression. We noted that codon optimality, bias and usage play a critical role in translation and mRNA decay (52). Codon usage score by GenScript showed a codon adaptable index of only 0.6 for VP64, indicating a poor codon usage. Thus, we selected reported sequences encoding VP64 (8,18,43) and generated codon-optimized VP64 variants by tools from GENEWIZ, Benchling, and jCAT. Screened through Codon usage score by GenScript, the highest scored VP64 coding sequence was used to replace the original version in V–D and minimized V–D (VPR-S-HNH-d1). The codon-optimized fusion proteins (indicated by asterisks) showed obviously increased protein expression and comparable gene activation for *IL1RN* in human cells and *Fgf21* in mouse cells in transient transfection experiments (Figure 4E). The codon-optimized VPR-S-HNH-d1 was a miniCas9 activator for gene expression and thus was termed as miniCAFE. To promote nuclear translocation of V–D, we fused one of the three strong nuclear localization signals (NLSs), a bipartite Ty1 retrotransposon NLS (Ty1NLS) (53), a bipartite nucleoplasmin NLS (NPM NLS) (53), and a bipartite SV40 NLS (bpNLS) (54,55), at the N- or C-terminus of VPR-d*Cj*Cas9 or replaced the 2xSV40 NLS between VPR and d*Cj*Cas9. Immunofluorescence staining and quantitative RT-PCR assays showed efficient nuclear translocation for most fusion proteins and comparable gene activation efficiency, except the ones with an N-terminal fused NLS that induced weak gene activation (Supplementary Figure S5A, B).

Further, we tried to optimize sgRNA to increase gene activation efficiency. Generally, transcription of sgRNA is controlled by the human U6 promoter, which prefers a guanine (G) for efficient transcription initiation (56). And thus the mature sgRNA is a GN_20_ structure with a mismatch at the 5′-end for *Sp*Cas9, which significantly decreases genome editing efficiency for high-fidelity *Sp*Cas9 (57). In our previous experiments, we generally used a GN_22_ sgRNA, which exhibited potent activity. To test whether elimination of the extra G would increase editing efficiency, we employed endogenous tRNA processing system to produce the exact matching sgRNA (57). However, it turned out to be no help (Supplementary Figure S5C). Next, we optimized the sgRNA scaffold of *Cj*Cas9 according to the reported strategies for that of *Sp*Cas9 (58,59). RT-PCR assay revealed significant enhancement for sgRNA^(F)^ and sgRNA^(FE)^, which were mutants with a A–U pair flip to eliminate of a putative type III polymerase termination signal and a further 5-bp extension of the hairpin to stabilize the *Cj*Cas9/sgRNA complex (Figure 4F and Supplementary Figure S5D).

Finally, we combined all the above effective strategies into a system, the miniCAFE/sgRNA^(F)^ system. For transient transfection experiments, we also optimized the mole ratio of transfected plasmids for d*Cj*Cas9 and sgRNA and found that 1:8 to 1:16 was about the optimal d*Cj*Cas9:sgRNA ratio for gene activation with either V-D or miniCAFE systems, in both human and mouse cells (Figure 4G). And we also noted that miniCAFE/sgRNA^(F)^ system showed more potent capability to activate gene expression in mouse cells at each ratio (Figure 4G). Again, RNA-seq revealed high specificity of the miniCAFE/sgRNA^(F)^ system and comparable gene expression profile between miniCAFE and V–D systems (Figure 4H, I).

### Gene activation with a single DNA copy of VPR-d*Cj*Cas9 and miniCAFE in mammalian cells

As previous data were based on transient transfection experiments, we next tested whether VPR-d*Cj*Cas9 and miniCAFE could work with a single DNA copy in mammalian cells. Using our currently developed high efficient knock-in (KI) method (60), we inserted a single copy of VPR-d*Cj*Cas9 and miniCAFE expression cassette into the *ACTB* site in MCF7 cells (Supplementary Figure S6A). KI clones were screened out by genomic PCR and two independent clones for each system were used for function characterization (Supplementary Figure S6B). The mRNA and protein expression level of miniCAFE was obviously higher than that of VPR-d*Cj*Cas9 (Supplementary Figure S6C, D). Consistently, when the cell clones were transfected with a sgRNA1^(F)^ and a sgRNA^(F)^ targeting the promoter regions of *IL1RN* and *NKX2.1*, respectively, the two genes were both potently activated and miniCAFE exhibited a stronger capability than VPR-d*Cj*Cas9 (Supplementary Figure S6E).

### Gene activation and corresponding phenotype induction in *C. elegans* with miniCAFE

**Figure 5.**
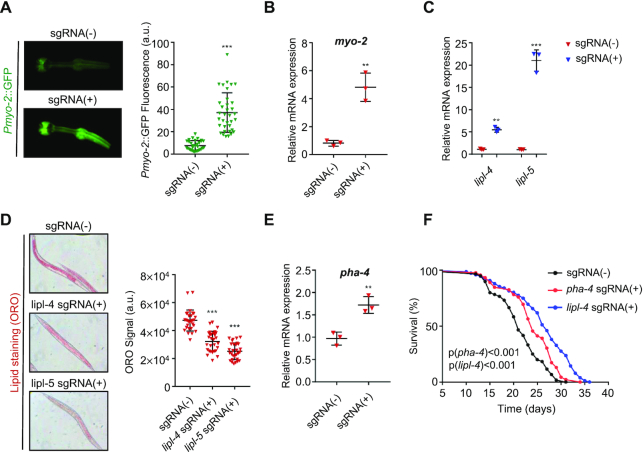
Targeted gene activation and corresponding phenotype induction in *C. elegans* with miniCAFE. (**A**) GFP fluorescence in the head region of the strains bearing *pdpy-30*::miniCAFE and *pmyo-2::GFP* transgenes in the presence or absence of the sgRNA targeting the *myo-2* promoter. (**B**) *myo-2* mRNA levels in the same worms as in A. (**C**) *lipl-4* and *lipl-5* mRNA levels in the miniCAFE transgenic worms in the presence or absence of the sgRNAs targeting the *lipl-4* and *lipl-5* promoters, respectively. (**D**) Oil Red O (ORO) staining in the same worms as in C. (**E**) *pha-4* mRNA levels in the miniCAFE transgenic worms in the presence or absence of the sgRNAs targeting the *pha-4* promoters. (**F**) Extended lifespan in in the miniCAFE transgenic worms in the presence of the sgRNA targeting the *lipl-4* or *pha-4* promoter. For A & D, data are mean ± SEM, *n* ≥ 30. For B, C and E, qRT-PCR revealed relative mRNA expression and data are mean ± S.D., *n* = 3 independent experiments. For **A–E**, **P < 0.01, ***P < 0.001 (Student's *t*-test, +sgRNA versus –sgRNA).

*C. elegans* has been a powerful model in a variety of studies. Using the CRISPR/Cas9 system, editing the genome has become a normal practice (61–64). Meanwhile, previous studies have shown that the CRISPR/Cas9-based transcription activator could up-regulate endogenous genes in *C. elegans* using catalytically inactive *Sp*Cas9 (65,66). To examine whether the *Cj*Cas9-based miniCAFE could function in *C. elegans*, we generated the P*dpy-30*::miniCAFE transgenic worm, in which miniCAFE could be expressed in all tissues as driven by the ubiquitous (*dpy-30*) promoter. When the transgenic animal was generated by co-injection with a pharyngeal fluorescence-bearing plasmid (P*myo-2*::GFP::H2B), injection with a third plasmid encoding a sgRNA targeting the *myo-2* promoter clearly increased the expression of GFP in pharyngeal muscle cells (Figure 5A). Quantification of the fluorescence intensity and RT-PCR assay both indicated an around 4-fold increase (Figure 5A, B). To test if miniCAFE could up-regulate endogenous genes, we chose three genes whose up-regulation caused clear phenotypes. *Lipl-4* and *lipl-5*, two lysosomal acid lipase genes, have been reported to regulate the lipid storage and longevity (67–69). The two genes were obviously activated by miniCAFE and the fat storage was decreased revealed by the Oil Red O (ORO) staining (Figure 5C, D). Similar to *lipl-4*, overexpression of *pha-4* has been reported to extend lifespan in *C. elegans* (67,70). As predicted, miniCAFE was able to activate these two genes and extend lifespan in the nematode (Figure 5C, E and F). Together, these results demonstrated that miniCAFE was able to activate endogenous genes and cause corresponding phenotypes in *C. elegans*, indicating a broader application in other functional studies when up-regulation of specific genes is required.

### Gene activation with VPR-d*Cj*Cas9 and miniCAFE in mice

Finally, we tested the VPR-d*Cj*Cas9 (V–D) and miniCAFE systems in mice. Fibroblast growth factor 21 (FGF21) is highly synthesized in the liver and plays a key role in energy homeostasis (71,72). Several studies have proven that FGF21 stimulates the brown (BAT) and beige adipose tissue thermogenesis, which lowers body weight and improves insulin sensitivity in mice (73,74). It has also been reported that the *Fgf21* promoter could be demethylated by CRISPR/dCas9-mediated epigenome editing (75). Therefore, we tried to activate FGF21 in mouse liver to regulate energy homeostasis. The AAV2/8 hybrid vector with liver-specific tropism was used to deliver the two systems.

The V–D fusion protein and sgRNA were packaged in separate AAVs since the size exceeded AAV capacity if packaged in a single AAV (Supplementary Figure S7A). C57BL/6 mice were intravenously injected with two AAV particles encoding V-D and sgFgf21/sgGFP. The body weight of AAV-FGF21-injected micewas lower than that of AAV-GFP-injected ones during a 79-day monitoring period although statistical significance was only observed at day 21 and day 48 (Supplementary Figure S7B). Of note, AAV-FGF21-injected mice exhibited improved systemic insulin sensitivity (Supplementary Figure S7C). Whole body indirect calorimetry analyses revealed augmented VO_2_ and VCO_2_, indicating increased energy expenditure (Supplementary Figure S7D, E). Consistently, RT-PCR assay confirmed the increased expression of important thermogenic genes, such as *Pgc1α* and *Pparα*, in subcutaneous white adipose tissue (scWAT) (Supplementary Figure S7F). UCP1 protein levels in scWAT and BAT were also elevated in AAV-FGF21-injected mice compared to AAV-GFP-injected ones (Supplementary Figure S7G).

**Figure 6.**
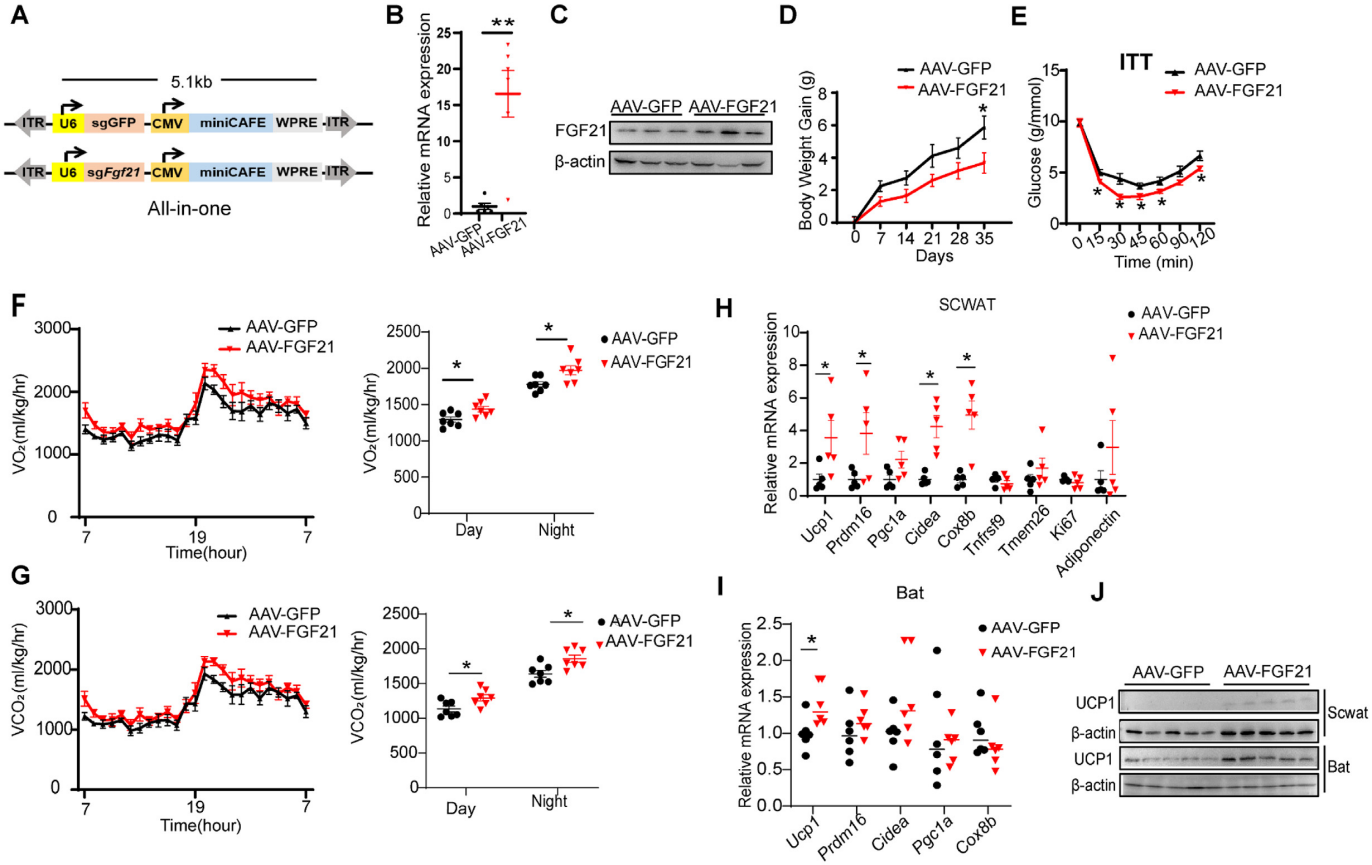
Metabolic regulation by activation of *Fgf21* in mouse liver with miniCAFE delivered with an all-in-one AAV vector. (**A**) The schematic of AAV-GFP and AAV-FGF21 all-in-one constructs, which contained a miniCAFE expression cassette and a sgRNA targeting GFP or targeting mouse FGF21 promoter region, respectively. (B, C) Fgf21 mRNA (**B**, n = 5) and FGF21 protein (**C**, n = 3) expression in liver of male C57BL/6 mice, which were intravenously injected with AAV-GFP or AAV-FGF21 through the tail vein. (D, E) Body weight gain (**D**) and insulin tolerance test (**E**) of AAV-GFP or AAV-FGF21 injected mice (*n* = 6). (F, G) Oxygen consumption (VO_2_) (**F**) and carbon dioxide production (VCO_2_) (**G**) of AAV-GFP or AAV-FGF21 injected mice (*n* = 7). (H, I) Representative thermogenic gene expression in scWAT (**H**, *n* = 5) and BAT (**I**, *n* = 6) of AAV-GFP or AAV-FGF21 injected mice. (**J**) UCP1 protein levels in scWAT and BAT of AAV-GFP or AAV-FGF21 injected mice (*n* = 5). Data are mean ± SEM. *P < 0.05, **P < 0.01 (Student's *t*-test).

As miniCAFE is smaller and more potent to activate transcription than V-D, we packaged miniCAFE and sgRNA in a single AAV (All-in-one) to induce FGF21 expression *in vivo* (Figure 6A). Elevated mRNA and protein expression was observed in AAV-FGF21-injected mice (Figure 6B, C). As a consequence, miniCAFE repressed body weight gain, improved systemic insulin sensitivity, increased energy expenditure, and increased expression of critical thermogenic genes in scWAT and BAT (Figure 6D–J).

Of note, Fgf21-induced phenotypic effects seem minor with significant differences at sporadic time points in the assays of body weight gain and insulin sensitivity (Figure 6 and Supplementary Figure S7). Several potential factors might account for this phenomenon. FGF21 has a relatively short half-life (from 0.5 to 2 h) and its concentration in the blood stream could be easily diminished through glomerular filtration in the kidney and by proteolytic degradation and aggregation (76,77). FGF21 has also been reported to increase food intake through its function in the brain (78,79), which may compromise the phenotypic effects by inducing more food intake. Finally, previous work has suggested that ‘FGF21 resistance’, downregulation of β-klotho (the obligate FGF21 co-receptor) in adipose tissue, is generally concomitant to increased circulating FGF21 level (80), which may also be a potential factor. Nevertheless, all the above results demonstrated that our VPR-d*Cj*Cas9 and miniCAFE systems activated gene transcription and caused corresponding phenotypes in mice.

## DISCUSSION

Collectively, based on *Cj*Cas9, we create a minimal target gene activator, miniCAFE, which is able to potently activate genes of interest and cause intended phenotypes in *C.elegans*, postnatal mice and human cells.

The miniCAFE system possesses several advantages. Foremost, it is small enough to be packaged within a single AAV particle. The all-in-one AAV approach not only increases delivery efficiency and thus gene activation efficiency but also decreases the cost. Secondly, it is a potent gene activator, as a single DNA copy of miniCAFE is able to robustly activate target genes even guided with a single sgRNA (Supplementary Figure S6) and the all-in-one AAV delivered miniCAFE induced robust FGF21 expression in the liver of postnatal mice (Figure 6). In previous reports, the large *Sp*Cas9-based gene activation system has to be packaged in two separate AAVs, which decreases gene activation efficiency (13,15,48). Alternatively, to be packaged within a single AAV, the relatively small d*Sa*Cas9 has to be fused with a very small transcriptional activator VP64 and to be packaged within a single sgRNA, which dramatically compromises the functionality, even worse than the two-separate-AAV system consisting of the d*Sa*Cas9-VP64 fusion protein and three sgRNAs (14). Therefore, our miniCAFE system might provide an alternative solution for the bottleneck of the size limitation. Finally, miniCAFE functions in worm, mice and human cells, implying a broad portability across disparate systems and species. Therefore, it is likely that miniCAFE holds potential as universal genetic tool to activate genes in various model and non-model organisms, like fish (zebrafish), frog (xenopus), and plant (arabidopsis), which needs to be tested further, of course.

With the advantages, miniCAFE holds great potential to treat human diseases. Via activating endogenous genes, *Sp*Cas9- and *Sa*Cas9-based gene activators have been reported to treat several diseases in mouse models (13–15). Additionally, via genome editing, *Cj*Cas9 has been reported to treat Duchenne muscular dystrophy (DMD) and to inhibit pathological choroidal neovascularization in mouse (19,22,23). More importantly, no severe long-term side effects are observed in the mouse when *Hif1a* in the retina is targeted by *Cj*Cas9 (23). Therefore, we believe that miniCAFE, a *Cj*Cas9-based gene activator, could be used to treat diseases as well. Of note, it is not always the case that the more the genes are expressed, the better the diseases are treated. To treat halploinfufficiency-caused disease, activating the functional copy to physiological expression level might be required. On the other side, to activate a modifier gene, a stronger activator might be required instead. Different sgRNAs targeting the promoter region could induce different expression levels. Alternatively, as shown in Figure 4B, more short transcription factors exhibit less potent activity, and when VPR-S is replaced by these factors, the gene activator can be easily packaged within AAVs and induce mild gene expression. However, similar to other CRISPR-based therapies, several potential risks might delay the clinical application of miniCAFE, such as the unknowing toxicity of *Cj*Cas9, potential immune responses and potential off-target effects.

Several factors could affect gene expression activated by *Cj*Cas9-based gene activators. Different gene activators, like SunTag systems, VPR systems and other systems, hold different capability to activate gene expression (Figures 1, 2, 4, and Supplementary Figures S1, S5 and S6). For a given gene, different gRNAs targeting different sites activate gene expression to different extent (Figures 1D, 2D, and Supplementary Figures S2C/D, S3C/D and S4E). Multiple gRNAs are generally more potent to activate gene expression than a single gRNA (Figures 1, 2, and Supplementary Figures S2 and S3). And cell context could also affect gene expression (Figures 1, 2, and Supplementary Figures S2 and S3). Therefore, owing to the complicated genetic and epigenetic background of each gene, it is necessary and essential to optimize the targeting sites and the combination of gRNAs in order to activate gene expression in a specific tissue with miniCAFE.

Apart from gene activation, d*Cj*Cas9 holds the potential to be used for repression of gene expression. For d*Sp*Cas9 and d*Sa*Cas9, both have been reported to be engineered for gene repression by blocking RNA polymerase elongation or by recruiting transcription repressors or domains (like KRAB, SID4X and the ZIM3 KRAB domain) (7,81). In theory, we believe that d*Cj*Cas9 would function in a similar way although further evaluation experiments are needed.

The PAM of *Cj*Cas9 is reported to be NNNVRYM (20) or NNNNRYAC (19) (V is A/G/C; R is A/G, Y is T/C; M is A/C). We find that sgRNAs with the PAM NNNNACAC generally induce potent gene activation. Compared to the NGG PAM of *Sp*Cas9, the potential targeting site of *Cj*Cas9 is more restricted. Thus, engineering PAM-flexible *Cj*Cas9 would further improves the miniCAFE system.

## DATA AVAILABILITY

RNA-seq and Deep-seq data are deposited on GEO database with GSE164452.

## Supplementary Material

gkab174_Supplemental_File
