# Erratum for article ‘MiniCAFE, a CRISPR/Cas9-based compact and potent transcriptional activator, elicits gene expression *invivo*’

**DOI:** 10.1093/nar/gkab365

**Published:** 2021-05-01

**Authors:** Xin Zhang, Sihan Lv, Zhenhuan Luo, Yongfei Hu, Xin Peng, Jie Lv, Shanshan Zhao, Jianqi Feng, Guanjie Huang, Qin-Li Wan, Jun Liu, Hongxin Huang, Bing Luan, Dong Wang, Xiaoyang Zhao, Ying Lin, Qinghua Zhou, Zhen-Ning Zhang, Zhili Rong

**Affiliations:** Cancer Research Institute, School of Basic Medical Sciences, Southern Medical University, Guangzhou 510515, China; Department of Endocrinology, Shanghai Tenth People's Hospital, School of Medicine, Tongji University, Shanghai 200072, China; Zhuhai Institute of Translational Medicine, Zhuhai People's Hospital Affiliated with Jinan University, Jinan University, Zhuhai 519000, China; The Biomedical Translational Research Institute, Faculty of Medical Science, Jinan University, Guangzhou 510632, China; Department of Bioinformatics, School of Basic Medical Sciences, Southern Medical University, Guangzhou 510515, China; Dermatology Hospital, Southern Medical University, Guangzhou 510091, China; Cancer Research Institute, School of Basic Medical Sciences, Southern Medical University, Guangzhou 510515, China; Cancer Research Institute, School of Basic Medical Sciences, Southern Medical University, Guangzhou 510515, China; Cancer Research Institute, School of Basic Medical Sciences, Southern Medical University, Guangzhou 510515, China; Cancer Research Institute, School of Basic Medical Sciences, Southern Medical University, Guangzhou 510515, China; Cancer Research Institute, School of Basic Medical Sciences, Southern Medical University, Guangzhou 510515, China; Zhuhai Institute of Translational Medicine, Zhuhai People's Hospital Affiliated with Jinan University, Jinan University, Zhuhai 519000, China; The Biomedical Translational Research Institute, Faculty of Medical Science, Jinan University, Guangzhou 510632, China; Dermatology Hospital, Southern Medical University, Guangzhou 510091, China; Dermatology Hospital, Southern Medical University, Guangzhou 510091, China; Department of Endocrinology, Shanghai Tenth People's Hospital, School of Medicine, Tongji University, Shanghai 200072, China; Department of Bioinformatics, School of Basic Medical Sciences, Southern Medical University, Guangzhou 510515, China; Dermatology Hospital, Southern Medical University, Guangzhou 510091, China; Department of Development, School of Basic Medical Sciences, Southern Medical University, Guangzhou 510515, China; Cancer Research Institute, School of Basic Medical Sciences, Southern Medical University, Guangzhou 510515, China; Zhuhai Institute of Translational Medicine, Zhuhai People's Hospital Affiliated with Jinan University, Jinan University, Zhuhai 519000, China; The Biomedical Translational Research Institute, Faculty of Medical Science, Jinan University, Guangzhou 510632, China; Translational Medical Center for Stem Cell Therapy & Institute for Regenerative Medicine, Shanghai East Hospital, School of Life Sciences and Technology, Tongji University, Shanghai 200092, China; Cancer Research Institute, School of Basic Medical Sciences, Southern Medical University, Guangzhou 510515, China; Dermatology Hospital, Southern Medical University, Guangzhou 510091, China; Bioland Laboratory (Guangzhou Regenerative Medicine and Health Guangdong Laboratory), Guangzhou 510005, China

The affiliations of Zhili Rong should be 1,6,9 not 1,4,9.

The article has been updated (1).
