# Supplementary material for: MiniCAFE, a CRISPR/Cas9-based compact and potent transcriptional activator, elicits gene expression in vivo
Source: Nucleic Acids Res. 2021 Mar 22;49(7):4171–85. doi: 10.1093/nar/gkab174 (PMC8053112; doi:10.1093/nar/gkab174)
Supplement: gkab174_Supplemental_File [file gkab174_supplemental_file.pdf]

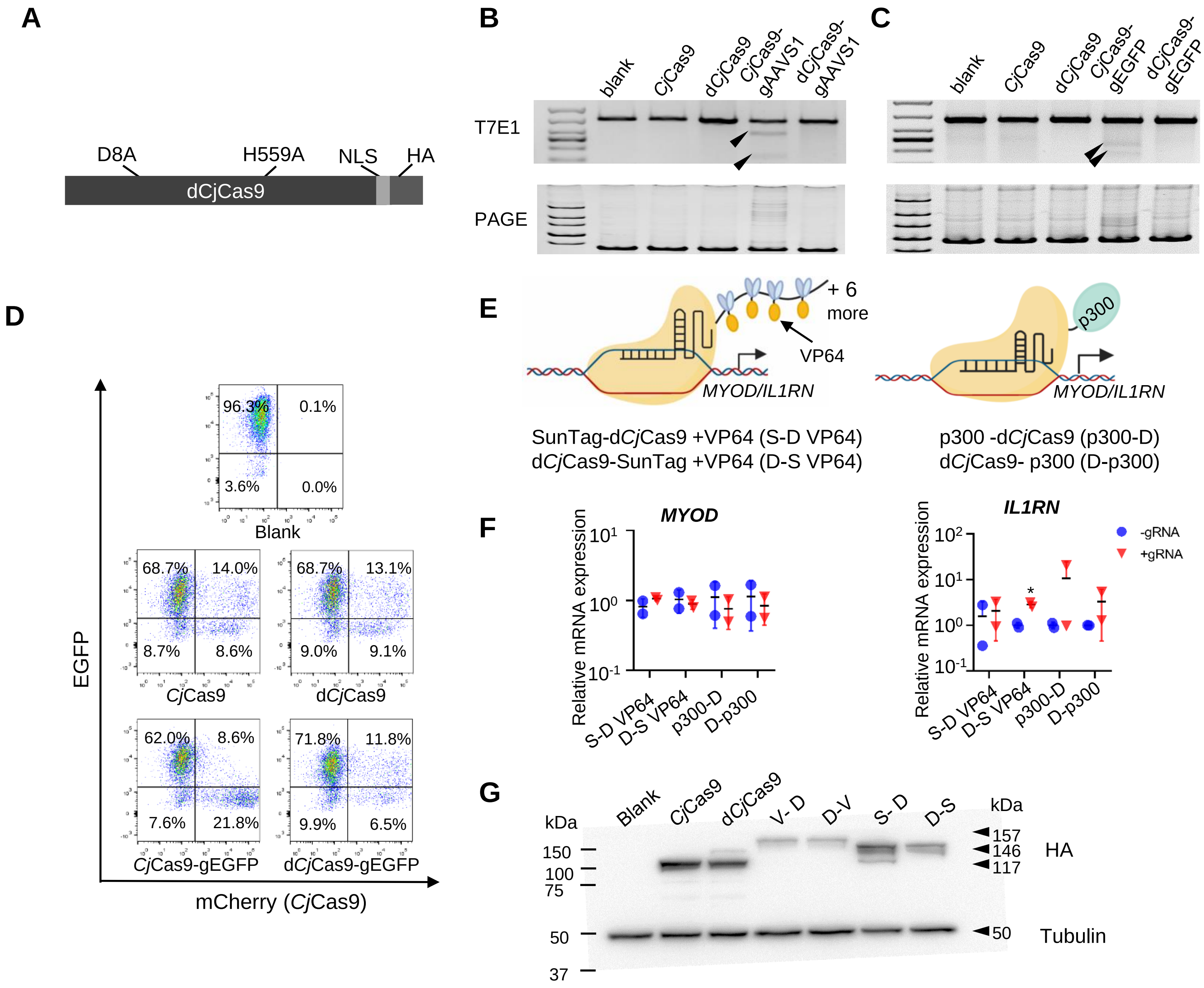

Supplementary Figure 1 Generation of d*Cj*Cas9 without DNase activity

Figure S1. Generation of d*Cj*Cas9 without DNase activity. (**A**) Schematic of d*Cj*Cas9. (**B**, **C**) T7E1 and PAGE analyses showing loss of DNase activity of d*Cj*Cas9. DNA was harvested from HEK293T cells co-transfected with wide-type *Cj*Cas9 or d*Cj*Cas9 and with a sgRNA targeting the AAVS1 site (**B**) or from a d2EGFP-knockin HEK293T reporter cells with a sgRNA targeting EGFP (**C**). Arrow heads indicate cleaved bands. (**D**) FACS analysis showing loss of DNase activity of d*Cj*Cas9. The d2EGFP-knockin HEK293T reporter cells were co-transfected with *Cj*Cas9/d*Cj*Cas9-P2A-mCherry and a sgRNA targeting EGFP, and harvested for FACS analysis 48 h after transfection. (**E**) Schematics of d*Cj*Cas9-SunTag-VP64 and d*Cj*Cas9-p300 activators. (**F**) Relative mRNA expression of *MYOD* and *IL1RN* revealed by qRT-PCR. HEK293T cells were co-transfected with indicated activators and four sgRNAs targeting each gene. Mean values are presented with S.D., n=2 independent experiments. * $p<0.05$ (Student's t-test, tested sample VS control sample). (**G**) Western blot of d*Cj*Cas9 fusion proteins in transfected HEK293T cells. V-D, VPR-d*Cj*Cas9; D-V, d*Cj*Cas9-VPR; S-D, SunTag-d*Cj*Cas9; D-S, d*Cj*Cas9-SunTag.

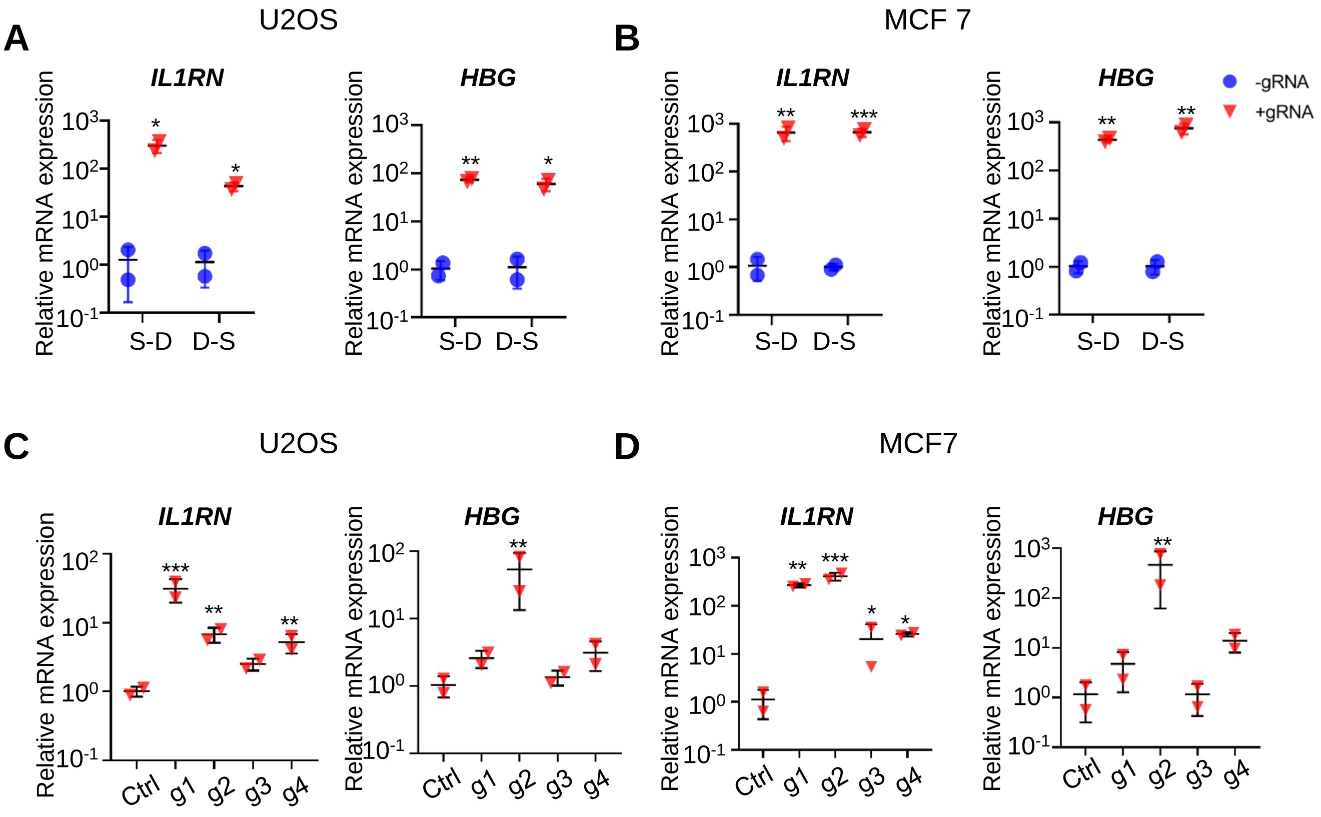

Supplementary Figure 2 Gene activation by the d*Cj*Cas9-SunTag-VPR system in multiple cell lines

Figure S2. Gene activation by the d*Cj*Cas9-SunTag-VPR system in multiple cell lines. (**A**, **B**) Relative mRNA expression of *IL1RN* and *HBG* in human bone osteosarcoma cells (U2OS) and human breast cancer cells (MCF7) co-transfected with S-D or D-S activators and four gRNAs targeting each gene. (**C**, **D**) Relative mRNA expression of *IL1RN* and *HBG* in U2OS and MCF7 cells co-transfected with S-D plasmid and four single gRNAs targeting each gene. For a-d, mean values are presented with S.D., n=2 independent experiments. *p<0.05, **p<0.01, ***p<0.001 (Student's t-test for A and B, one-way ANOVA test for C and D, tested sample VS control sample).

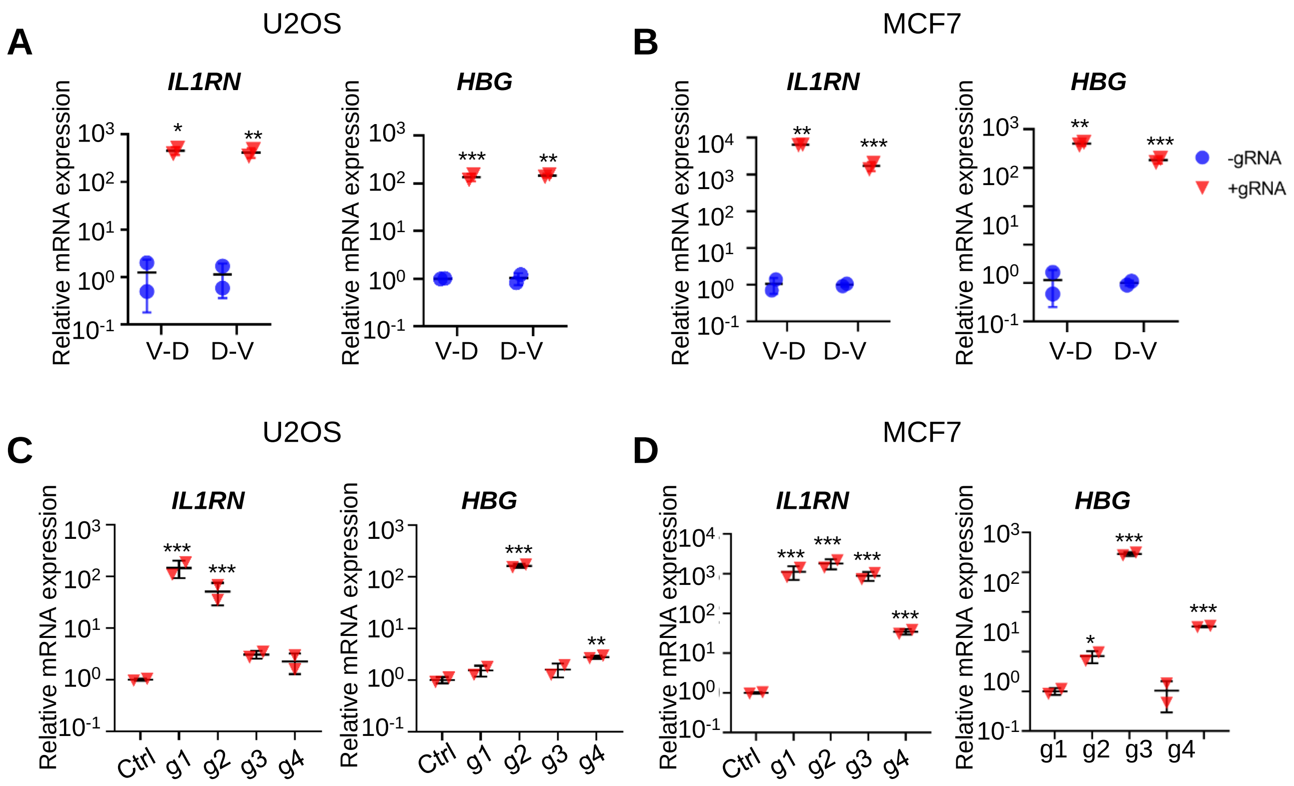

Supplementary Figure 3 Gene activation by the VPR-d*Cj*Cas9 system in multiple cell lines

Figure S3. Gene activation by the VPR-d*Cj*Cas9 system in multiple cell lines. (**A**, **B**) Relative mRNA expression of *IL1RN* and *HBG* in U2OS and MCF7 cells co-transfected with V-D or D-V plasmids and four gRNAs targeting each gene. (**C**, **D**) Relative mRNA expression of *IL1RN* and *HBG* in U2OS and MCF7 co-transfected with V-D plasmid and four single gRNAs targeting each gene. For a-d, mean values are presented with S.D., n=2 independent experiments. *p<0.05, **p<0.01, ***p<0.001 (Student's t-test for A and B, one-way ANOVA test for C and D, tested sample VS control sample).

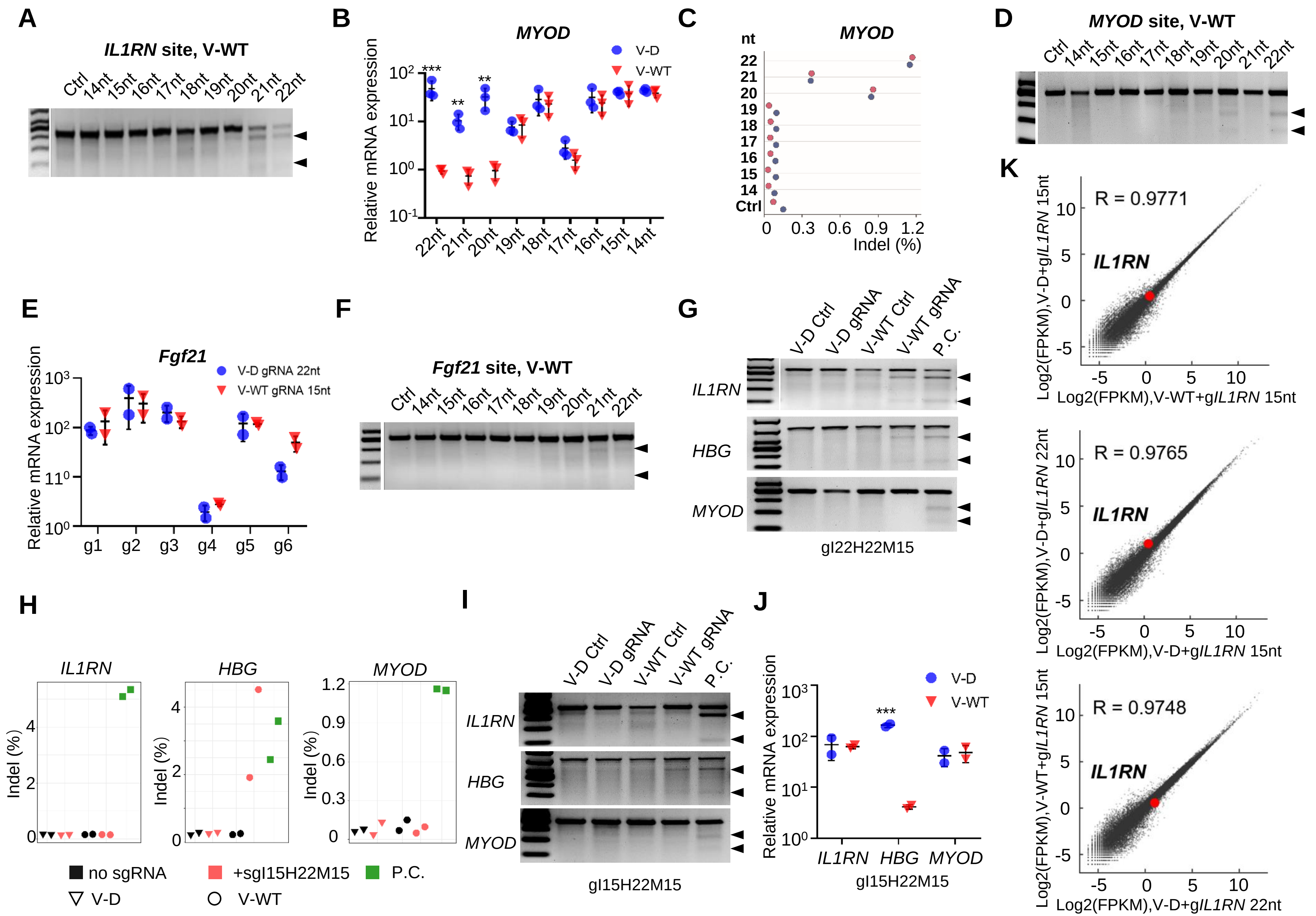

Supplementary Figure 4 Orthogonal genome editing and transcriptional activation with a VPR-*Cj*Cas9 fusion nuclease

Figure S4. Orthogonal genome editing and transcriptional activation with a VPR-*Cj*Cas9 fusion nuclease. (**A**) Genome editing with VPR-*Cj*Cas9 (V-WT) at the *IL1RN* site. (**B**) Relative mRNA expression of *MYOD*. (**C**, **D**) DNase activities of V-WT at the *MYOD* site. For **A**-**D**, HEK293T cells were co-transfected with either V-WT or V-D and the gRNAs with indicated length. (**E**) Relative mRNA expression of *Fgf21*. Mouse B16 cells were co-transfected with V-WT and six single 15-nt gRNAs targeting the *Fgf21* promoter or with V-D and six single 22-nt gRNAs targeting the same sites. (**F**) Genome editing with V-WT at the *Fgf21* site. (**G**) Indel formation with V-WT guided by gI22H22M15. (**H**-**J**) Multiplexed orthogonal genome editing at the *HBG* sites and gene activation of *IL1RN* and *MYOD*. gI15H22M15, a combination of three sgRNAs including a 15-nt g*IL1RN*, a 22-nt g*HBG*, and a 15-nt g*MYOD*. P.C., positive control, the same as that in Figure 3E. (**K**) The gene expression profile comparison between V-WT/g*IL1RN*-15nt, V-D/g*IL1RN*-15nt, and V-D/g*IL1RN*-22nt. Average of two biological replicates was shown. For **B**, **E**, and **J**, mean values are presented with S.D., n=2-3 independent experiments. **p<0.01, ***p<0.001 (Student's t-test, V-D sample VS corresponding V-WT sample). Genome editing was revealed by Deep-seq (**C**, **H**) and T7E1 assays (**A**, **D**, **F**, **G**, and **J**), and relative mRNA expression was revealed by qRT-PCR (B, E, and J)

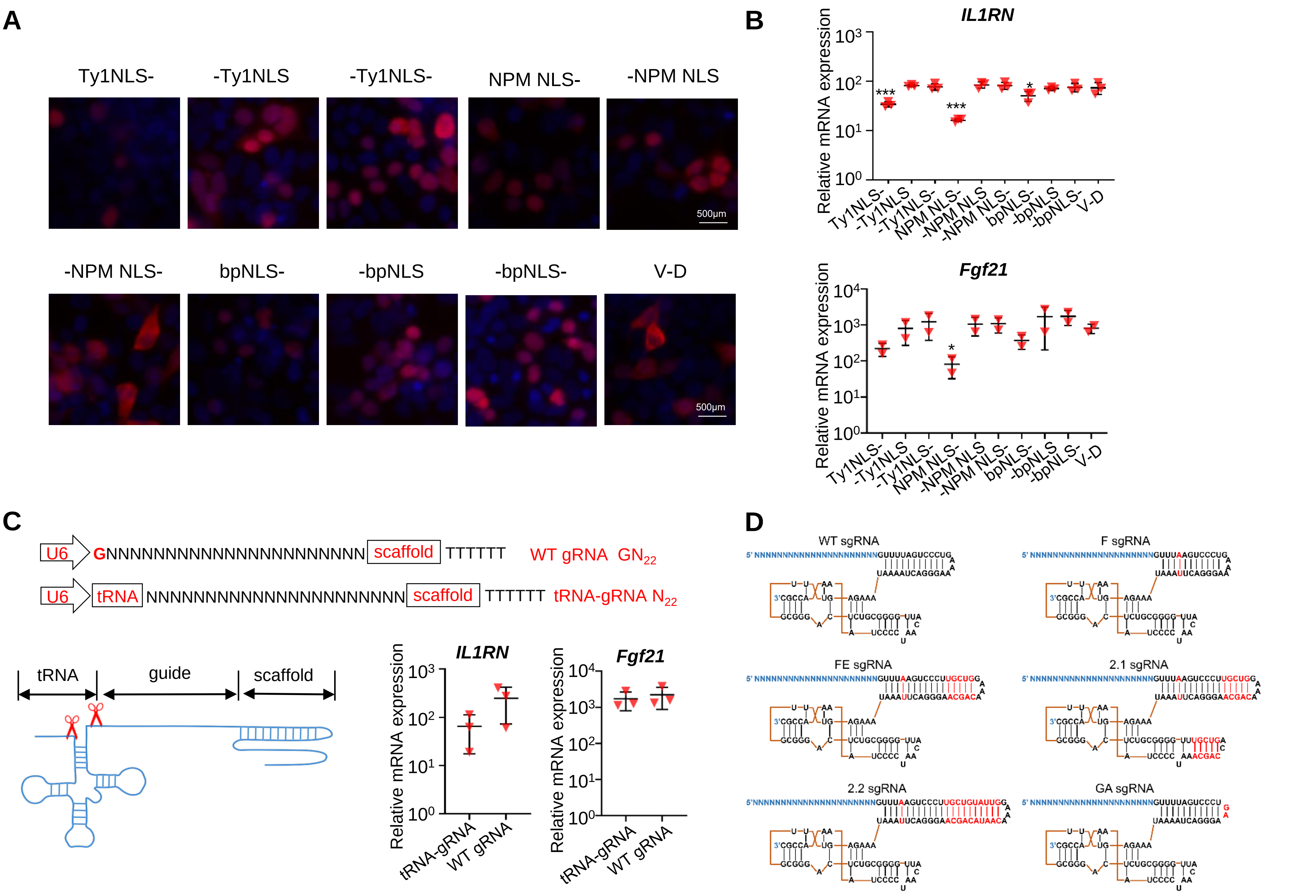

Supplementary Figure 5 Optimization of the VPR-d*Cj*Cas9 system

Figure S5. Optimization of the VPR-d*Cj*Cas9 system. (**A**) Immunofluorescence staining revealed nuclear localization of VPR-d*Cj*Cas9 proteins fused with different nuclear localization signals (NLSs). (**B**) qRT-PCR revealed relative mRNA expression of *IL1RN* and *Fgf21* in human HEK293T and mouse B16 cells, respectively. sgRNA1 for *IL1RN* and sgRNA2 for *Fgf21* were co-transfected with V-D proteins fused with indicated NLSs. NLS-, NLS was fused to the N-terminus of V-D. -NLS-, NLS was inserted between VPR and d*Cj*Cas9. -NLS, NLS was fused to the C-terminus of V-D. (**C**) qRT-PCR revealed relative mRNA expression of *IL1RN* and *Fgf21* in human HEK293T and mouse B16 cells, respectively. tRNA was fused upstream of sgRNA1 for *IL1RN* and sgRNA2 for *Fgf21* to facilitate sgRNA processing. (**D**) The schematic of sgRNA structures. For **B** and **C**, mean values are presented with S.D., n=2-3 independent experiments. *p<0.05, ***p<0.001 (One-way ANOVA test for **B**, tested sample VS V-D sample; Student's t-test. for **C**, tRNA-gRNA VS WT gRNA).

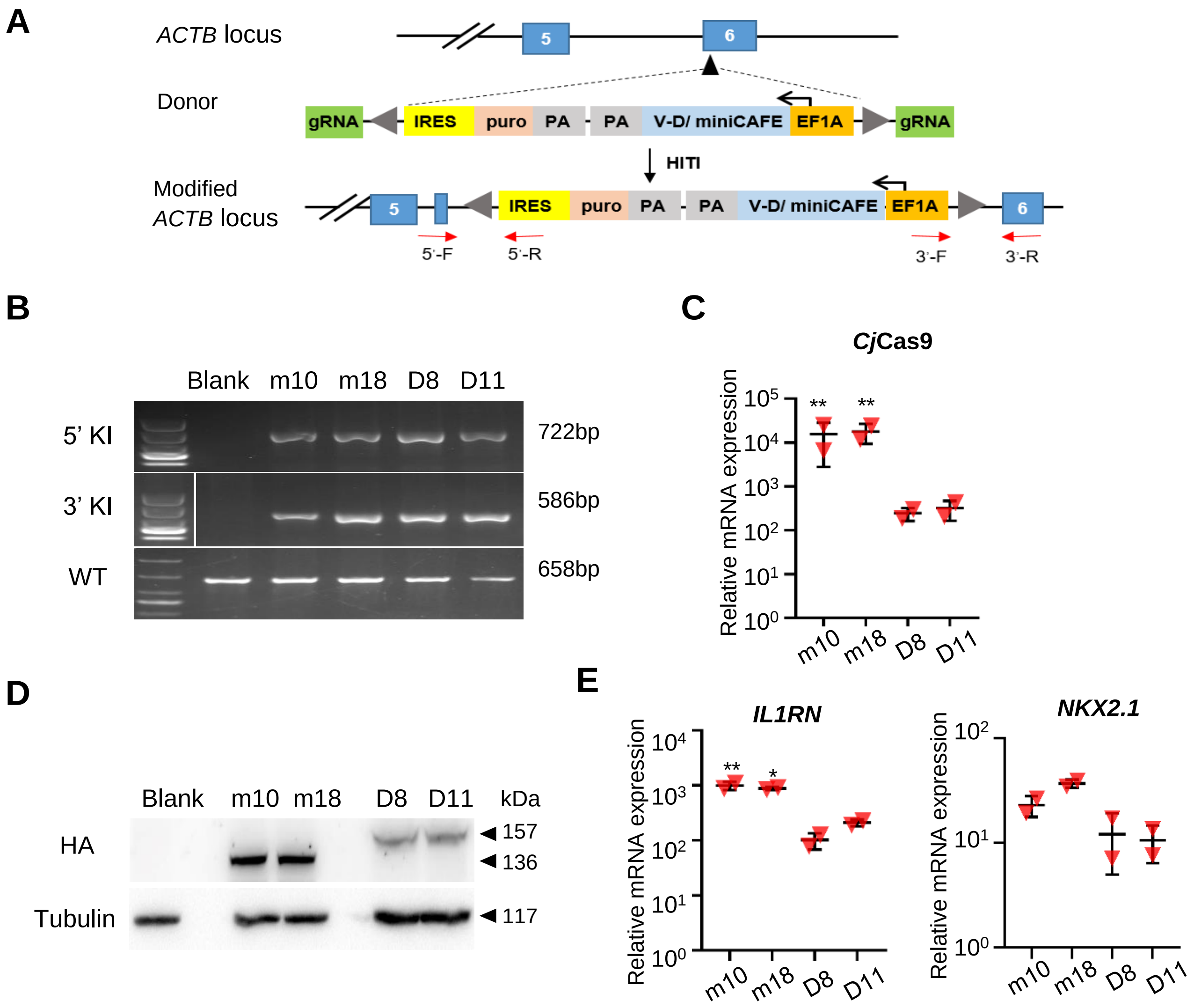

Supplementary Figure 6 Robust gene activation with a single copy of VPR-d*Cj*Cas9 and miniCAFE in mammalian cells

Figure S6. Robust gene activation with a single copy of VPR-d*Cj*Cas9 and miniCAFE in mammalian cells. (**A**) The schematic of knock-in strategy for V-D and miniCAFE using the Cas9-N57 nuclease. (**B**) Identification of knock-in (KI) clones by genomic PCR. m10 and m18, two independent miniCAFE clones; D8 and D11, two independent V-D clones. (**C**, **D**) The mRNA and protein expression level of miniCAFE and V-D in KI clones. (**E**) Activation of *IL1RN* and *NKX2.1* in miniCAFE and V-D KI clones. Cells were transfected with the gRNA1 for *IL1RN* and a sgRNA for *NKX2.1,* and RNA was harvested 48 h after transfection. For **C**, and **E**, mean values are presented with S.D., n=2 independent experiments. For each experiment, fold changes of mRNA expression in tested samples (with sgRNA) versus that in control samples (without sgRNA) were shown. *$p<0.05$, **$p<0.01$ (One-way ANOVA test, tested sample VS D11 sample).

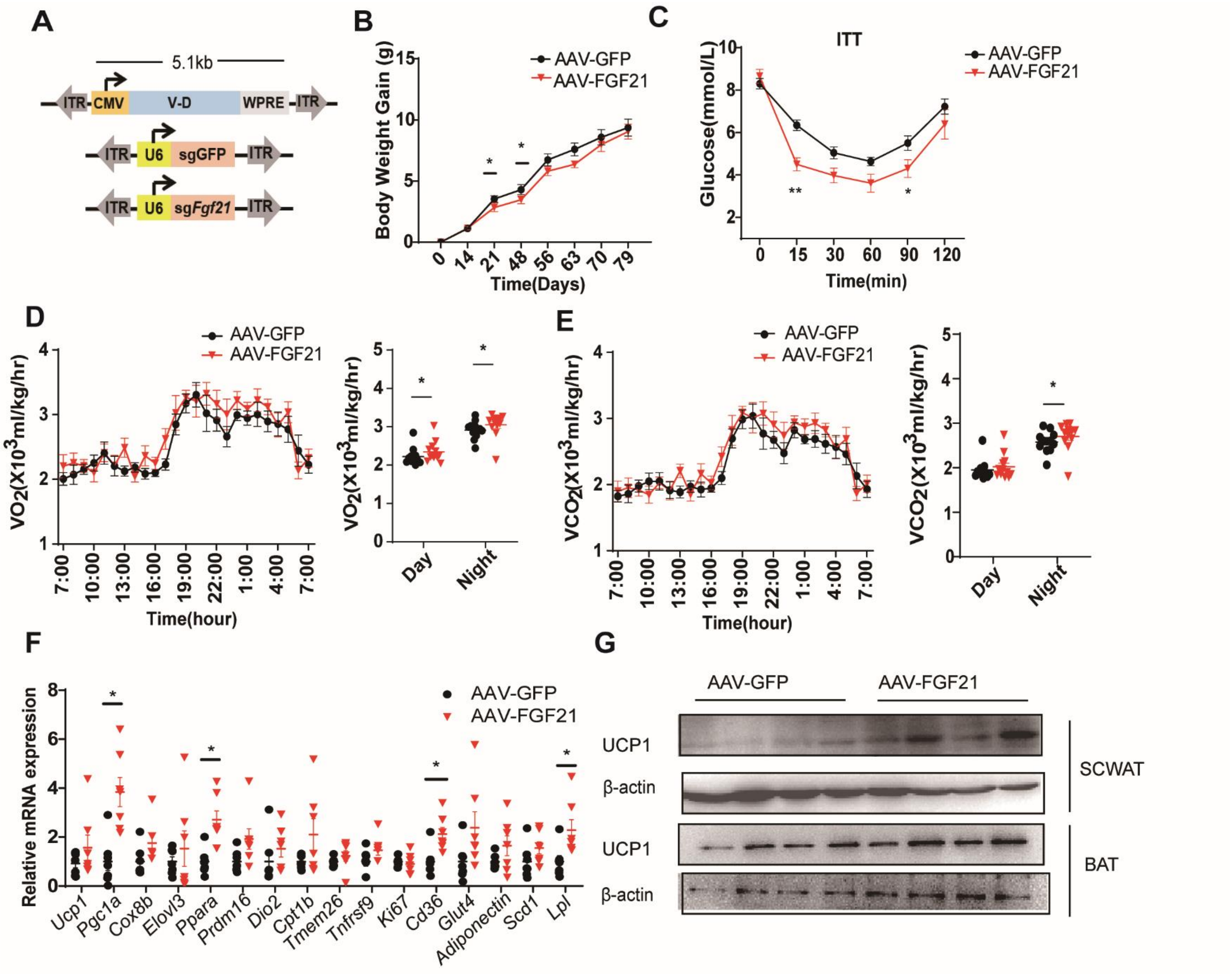

Supplementary Figure 7 Metabolic regulation by activation of *Fgf21* in mouse liver with the VPR-d*Cj*Cas9 system

Figure S7. Metabolic regulation by activation of *Fgf21* in mouse liver with the VPR-d*Cj*Cas9 system. (**A**) The schematic of AAV-VPR-d*Cj*Cas9 (V-D), AAV-gGFP and AAV-gFGF21, which encoded VPR-d*Cj*Cas9, a sgRNA targeting GFP and the sgRNA2 targeting mouse *Fgf21* promoter region, respectively. Male PVALB-KO mice were intravenously injected with AAV-V-D and AAV-gGFP (termed AAV-GFP) or with AAV-V-D and AAV-gFGF21 (termed AAV-FGF21) through the tail vein. (**B**, **C**) Body weight (**B**) and insulin tolerance test (**C**) of AAV-GFP or AAV-FGF21 injected mice (n=6). (**D**, **E**) Oxygen consumption (VO_2) (**D**) and carbon dioxide production (VCO_2) (**E**) of AAV-GFP or AAV-FGF21 injected mice (n=6). (**F**) Representative thermogenic gene expression in scWAT of AAV-GFP or AAV-FGF21 injected mice (n=6). (**G**) UCP1 protein levels in scWAT and BAT of AAV-GFP or AAV-FGF21 injected mice (n=4). Data are mean ± SEM. * $p<0.05$ (Student's t-test).

SupplementaryTable 1:

Primers for genotyping	*sequence (5'-3')*
5’-F	CCACCATGTACCCTGGCATT
5’-R	TAGATGTCCTAACTGACTTGCC
3’-F	AGAACGTTCACGGCGACTACT
3’-R	TGTGTGGACTTGGGAGAGGA
5'-KI (5'-F+5'-R), 3'-KI (3'-F+3'-R), WT (5’-F+3’-R)	

sgRNA targeting sites	*sequence (5'-3')*
GFP-gRNA	CCGCGCCGAGGTGAAGTTCGAG
AAVS1-gRNA	GTTAGGCAGATTCCTTATCTGG
MYOD-gRNA1	CGGCACGCCCTTTCCAAACCTC
MYOD-gRNA2	AGACTGGCCAGCCAGCCCGCCC
MYOD-gRNA3	GGAGGGGGAGTCCGAGGCCAAT
MYOD-gRNA4	GGAGCGACCGGCAGCCCTAGGC
IL1RN-gRNA1	TCCCAATGCCTGGAAGAGTGCC
IL1RN-gRNA2	ACTGCCGCCAGCTCATGCATGT
IL1RN-gRNA3	CAAGGAGGAGCCCAGCCTCCCC
IL1RN-gRNA4	AGTGCCTGGCACACAGGATTAA
HBG-gRNA1	CTAGTTTCCTTCTCCCATCATA
HBG-gRNA2	TCAAAAATCCTGGACCTATGCC
HBG-gRNA3	TTCCCTGAGAAGTGAACCTAGC
HBG-gRNA4	AGTATCCTCTTGGGGGCCCCTT
NKX2.1-gRNA	CAGAAGAGAGGCAGACAGACTG
Fgf21-gRNA1	CCCACTCCTGACGCGTGATATT
Fgf21-gRNA2	TCCCCACTCCTGACGCGTGATA
Fgf21-gRNA3	CCAACCCTCCTCCCTCAGACCA
Fgf21-gRNA4	GGGTCTGAGGTAGGAGACTGAA
Fgf21-gRNA5	CCTGCTGGGCTGAGGACTCCTC
Fgf21-gRNA6	GCTCTCCTGATGAAAGAAAGCC
myo-2-gRNA	GGGGATGTCACAATAAAACGTC
lipl-4-gRNA	GATTTGCACTTCACATACACAC
lipl-5-gRNA	AAGATAAGCTGTTTGGCGCTGT
aak-2-gRNA	AATATGTTCAGATGCTCGAGTG
pha-4-gRNA	TCTGTGCGAGACTATTAAAGTG

Primers for qPCR	*sequence (5'-3')*
MYOD-qPCR-F	CCGACGGCATGATGGACTAC
MYOD-qPCR-R	AGGCAGTCTAGGCTCGACAC
IL1RN-qPCR-F	GGAATCCATGGAGGGAAGAT
IL1RN-qPCR-R	TGTTCTCGCTCAGGTCAGTG
HBG-qPCR-F	GCTGAGTGAACTGCACTGTGA
HBG-qPCR-R	GAATTCTTTGCCGAAATGGA
NKX2.1-qPCR-F	CTCATGTTCATGCCGCTC
NKX2.1-qPCR-R	GACACCATGAGGAACAGCG
GAPDH-qPCR-F	AGAAGGCTGGGGCTCATTTG
GAPDH-qPCR-R	AGGGGCCATCCACAGTCTTC
CjCas9-qPCR-F	CCCGCCTTCAACGAAACCTA
CjCas9-qPCR-R	GTTGATCTTGTGCACCTTGC

Gapdh-qPCR-F1	GGTGAAGGTCGGTGTGAAC
Gapdh-qPCR-R1	GAGTGGAGTCATACTGGAAC
Actin-qPCR-F1	AACATCGAAGAGGACTTCCGA
Actin-qPCR-R1	CAAGCGTTCACCTGAGATGAC
Fgf21-qPCR-F	CAAGACACTGAAGCCCACCT
Fgf21-qPCR-R	CACCCAGGATTTGAATGACC
Ucp1-qPCR-F	ACTGCCACACCTCCAGTCATT
Ucp1-qPCR-R	CTTTGCCTCACTCAGGATTGG
Prdm16-qPCR-F	CAGCACGGTGAAGCCATTC
Prdm16-qPCR-R	GCGTGCATCCGCTTGTG
Pgc1a-qPCR-F	CCCTGCCATTGTTAAGACC
Pgc1a-qPCR-R	TGCTGCTGTTCCTGTTTTC
Cox8b-qPCR-F	GAACCATGAAGCCAACGACT
Cox8b-qPCR-R	GCGAAGTTCACAGTGGTTCC
Cidea-qPCR-F	TGCTCTTCTGTATCGCCCAGT
Cidea-qPCR-R	GCCGTGTTAAGGAATCTGCTG
Adiponectin-qPCR-F	GGAGAGAAAGGAGATGCAGGT
Adiponectin-qPCR-R	CTTTCCTGCCAGGGGTTC
Tmem26-qPCR-F	ACCCTGTCATCCCACAGAG
Tmem26-qPCR-R	TGTTTGGTGGAGTCCTAAGGTC
Tnfrsf9-qPCR-F	CGTGCAGAACTCCTGTGATAAC
Tnfrsf9-qPCR-R	GTCCACCTATGCTGGAGAAGG
Ki67-qPCR-F	ATCATTGACCGCTCCTTTAGGT
Ki67-qPCR-R	GCTCGCCTTGATGGTTCCT
Lpl-qPCR-F	ACTCTGTGTCTAACTGCCACTTCAA
Lpl-qPCR-R	ATACATTCCCGTTACCGTCCAT
Elovl3-qPCR-F	TTCTCACGCGGGTTAAAAATGG
Elovl3-qPCR-R	GAGCAACAGATAGACGACCAC
Ppara-qPCR-F	AGAGCCCCATCTGTCCTCTC
Ppara-qPCR-R	ACTGGTAGTCTGCAAAACCAAA
Dio2-qPCR-F	GTCCGCAAATGACCCCTTT
Dio2-qPCR-R	CCCACCCACTCTCTGACTTTC
Cpt1b-qPCR-F	GCTGCTTGCACATTTGTGTT
Cpt1b-qPCR-R	TGAGTGACTGGTGGGAAGAA
Cd36-qPCR-F	GGAACTGTGGGCTCATTGC
Cd36-qPCR-R	CATGAGAATGCCTCCAAACAC
Glut4-qPCR-F	GTGACTGGAACACTGGTCCTA
Glut4-qPCR-R	CCAGCCACGTTGCATTGTAG
Scd1-qPCR-F	TTCTTGCGATACACTCTGGTGC
Scd1-qPCR-R	CGGGATTGAATGTTCTTGTCGT
GFP(S65C)-qPCR-F	TCTGTCAGTGGAGAGGGTGA
GFP(S65C)-qPCR-R	GACAAGTGTTGGCCATGGAAC
lipl-4-qPCR-F	AAAACAAGACCTGGAAGAAACG
lipl-4-qPCR-R	ATAAACTTGGCTGGCTGCAT
lipl-5-qPCR-F	TCAGGATGTTGTGGGAAGCC
lipl-5-qPCR-R	GGCCATGTTACGTTTGTTTTCC
pha-4-qPCR-F	GCCAATTTCATGCAAGGAGG
pha-4-qPCR-R	GCCAGTGGTAAAACCAAGAGGT
cdc-42-qPCR-F	CTGCTGGACAGGAAGATTACG
cdc-42-qPCR-R	CTCGGACATTCTCGAATGAAG

Primers for T7E1	*sequence (5'-3')*
EGFP-T7E1-F	TGAAGTTCATCTGCACCACCG
EGFP-T7E1-R	TGTGATCGCGCTTCTCGTT
AAVS1-T7E1-F	GGACAACCCCAAAGTACCCC
AAVS1-T7E1-R	ACCAGGATCAGTGAAACGCA
MYOD-T7E1-F	TTCTACAGCCGCTCTACCCA
MYOD-T7E1-R	CCGTAGTAGCCTAAACGCCC
IL1RN-T7E1-F	TAGAGCGTTGGGGACCTTGT
IL1RN-T7E1-R	CATAAAGTAGCACCCGGGCA
HBG-T7E1-F	TAGCCTTTGCCTTGTTCCGA
HBG-T7E1-R	ACACGCACATCTTATGTCTTAGAG
mFgf21-T7E1-F	GTCCCAGGGTCCCAACTCTA
mFgf21-T7E1-R	CCCATGCCTAGCCCTTTTCA

Primers for PAGE	*sequence (5'-3')*
EGFP-PAGE-F	CACATGAAGCAGCACGACT
EGFP-PAGE-R	GGACTGGGTGCTCAGGTAGT
AAVS1-PAGE-F	CCGGTTAATGTGGCTCTGGT
AAVS1-PAGE-R	CTCCCTCCCAGGATCCTCTC

Primers for Deep-seq	*sequence (5'-3')*
IL1RN-DS-F1	acgCTCTTTCCCTACACGACGCTCTTCCGATCT*TAGAGC* TAGAGCGTTGGGGACCTTGT
IL1RN-DS-R2-1	actACTGGAGTTCAGACGTGTGCTCTTCCGATCT*CTTACG* GAACTGAGCTGGGCCTTCG
IL1RN-DS-R2-2	actACTGGAGTTCAGACGTGTGCTCTTCCGATCT*ATCACG* GAACTGAGCTGGGCCTTCG
IL1RN-DS-R2-3	actACTGGAGTTCAGACGTGTGCTCTTCCGATCT*CGATGT* GAACTGAGCTGGGCCTTCG
IL1RN-DS-R2-4	actACTGGAGTTCAGACGTGTGCTCTTCCGATCT*TTAGGC* GAACTGAGCTGGGCCTTCG
IL1RN-DS-R2-5	actACTGGAGTTCAGACGTGTGCTCTTCCGATCT*TGACCA* GAACTGAGCTGGGCCTTCG
IL1RN-DS-R2-6	actACTGGAGTTCAGACGTGTGCTCTTCCGATCT*ACAGTG* GAACTGAGCTGGGCCTTCG
IL1RN-DS-R2-7	actACTGGAGTTCAGACGTGTGCTCTTCCGATCT*GCCAAT* GAACTGAGCTGGGCCTTCG
IL1RN-DS-R2-8	actACTGGAGTTCAGACGTGTGCTCTTCCGATCT*CAGATC* GAACTGAGCTGGGCCTTCG
IL1RN-DS-R2-9	actACTGGAGTTCAGACGTGTGCTCTTCCGATCT*ACTTGA* GAACTGAGCTGGGCCTTCG
IL1RN-DS-R2-10	actACTGGAGTTCAGACGTGTGCTCTTCCGATCT*GATCAG* GAACTGAGCTGGGCCTTCG
IL1RN-DS-R2-11	actACTGGAGTTCAGACGTGTGCTCTTCCGATCT*TAGCTT* GAACTGAGCTGGGCCTTCG
IL1RN-DS-R2-12	actACTGGAGTTCAGACGTGTGCTCTTCCGATCT*GGCTAC* GAACTGAGCTGGGCCTTCG
IL1RN-DS-R2-13	actACTGGAGTTCAGACGTGTGCTCTTCCGATCT*CTTGTA* GAACTGAGCTGGGCCTTCG

IL1RN-DS-R2-14	actACTGGAGTTCAGACGTGTGCTCTTCCGATCT*AGTCAA* GAACTGAG CTGGGCCTTCG
IL1RN-DS-R2-15	actACTGGAGTTCAGACGTGTGCTCTTCCGATCT*AGTTCC* GAACTGAG CTGGGCCTTCG
IL1RN-DS-R2-16	actACTGGAGTTCAGACGTGTGCTCTTCCGATCT*ATGTCA* GAACTGAG CTGGGCCTTCG
IL1RN-DS-R2-17	actACTGGAGTTCAGACGTGTGCTCTTCCGATCT*CCGTCC* GAACTGAG CTGGGCCTTCG
IL1RN-DS-R2-18	actACTGGAGTTCAGACGTGTGCTCTTCCGATCT*ATACGA* GAACTGAG CTGGGCCTTCG
IL1RN-DS-R2-19	actACTGGAGTTCAGACGTGTGCTCTTCCGATCT*AATCGC* GAACTGAG CTGGGCCTTCG
MYOD-DS-R2	actACTGGAGTTCAGACGTGTGCTCTTCCGATCT*CTTGTA* TAGTAGCC TAAACGCCCCG
MYOD-DS-F1-1	acgCTCTTTCCCTACACGACGCTCTTCCGATCT*GTCCGC* GGAGAGCC AAGTGCAGCC
MYOD-DS-F1-2	acgCTCTTTCCCTACACGACGCTCTTCCGATCT*GTTTCG* GGAGAGCCA AGTGCAGCC
MYOD-DS-F1-3	acgCTCTTTCCCTACACGACGCTCTTCCGATCT*CGTACG* GGAGAGCC AAGTGCAGCC
MYOD-DS-F1-4	acgCTCTTTCCCTACACGACGCTCTTCCGATCT*GAGTGG* GGAGAGCC AAGTGCAGCC
MYOD-DS-F1-5	acgCTCTTTCCCTACACGACGCTCTTCCGATCT*ACTGAT* GGAGAGCCA AGTGCAGCC
MYOD-DS-F1-6	acgCTCTTTCCCTACACGACGCTCTTCCGATCT*TAGCTG* GGAGAGCCA AGTGCAGCC
MYOD-DS-F1-7	acgCTCTTTCCCTACACGACGCTCTTCCGATCT*TAAGCT* GGAGAGCCA AGTGCAGCC
MYOD-DS-F1-8	acgCTCTTTCCCTACACGACGCTCTTCCGATCT*GTCTTA* GGAGAGCCA AGTGCAGCC
MYOD-DS-F1-9	acgCTCTTTCCCTACACGACGCTCTTCCGATCT*GAGCGC* GGAGAGCC AAGTGCAGCC
MYOD-DS-F1-10	acgCTCTTTCCCTACACGACGCTCTTCCGATCT*CTGCGC* GGAGAGCC AAGTGCAGCC
MYOD-DS-F1-11	acgCTCTTTCCCTACACGACGCTCTTCCGATCT*GCAGCG* GGAGAGCC AAGTGCAGCC
MYOD-DS-F1-12	acgCTCTTTCCCTACACGACGCTCTTCCGATCT*AGGCTC* GGAGAGCC AAGTGCAGCC
MYOD-DS-F1-13	acgCTCTTTCCCTACACGACGCTCTTCCGATCT*GTAGCT* GGAGAGCCA AGTGCAGCC
MYOD-DS-F1-14	acgCTCTTTCCCTACACGACGCTCTTCCGATCT*TAGCGA* GGAGAGCCA AGTGCAGCC
MYOD-DS-F1-15	acgCTCTTTCCCTACACGACGCTCTTCCGATCT*CCTGAG* GGAGAGCC AAGTGCAGCC
MYOD-DS-F1-16	acgCTCTTTCCCTACACGACGCTCTTCCGATCT*TCATGA* GGAGAGCCA AGTGCAGCC
MYOD-DS-F1-17	acgCTCTTTCCCTACACGACGCTCTTCCGATCT*CCTCTA* GGAGAGCCA AGTGCAGCC
MYOD-DS-F1-18	acgCTCTTTCCCTACACGACGCTCTTCCGATCT*TGCATT* GGAGAGCCA AGTGCAGCC
MYOD-DS-F1-19	acgCTCTTTCCCTACACGACGCTCTTCCGATCT*CCAGAT* GGAGAGCCA AGTGCAGCC
MYOD-DS-F1-20	acgCTCTTTCCCTACACGACGCTCTTCCGATCT*ATTAGC* GGAGAGCCA AGTGCAGCC

MYOD-DS-F1-21	acgCTCTTTCCCTACACGACGCTCTTCCGATCT*GACATC* GGAGAGCCA AGTGCAGCC
MYOD-DS-F1-22	acgCTCTTTCCCTACACGACGCTCTTCCGATCT*TGACTT* GGAGAGCCA AGTGCAGCC
MYOD-DS-F1-23	acgCTCTTTCCCTACACGACGCTCTTCCGATCT*GGACTA* GGAGAGCCA AGTGCAGCC
HBG-DS-F2	cgCTCTTTCCCTACACGACGCTCTTCCGATCT*TCGGCA* TGTCTCTAGC TCCAGTGAGGC
HBG-DS-R1-1	ctACTGGAGTTCAGACGTGTGCTCTTCCGATCT*TCGAAG* TTTCCTTAG AAACCACTGCTAACT
HBG-DS-R1-2	ctACTGGAGTTCAGACGTGTGCTCTTCCGATCT*TCCCGA* TTTCCTTAG AAACCACTGCTAACT
HBG-DS-R1-3	ctACTGGAGTTCAGACGTGTGCTCTTCCGATCT*TCATTC* TTTCCTTAGA AACCACTGCTAACT
HBG-DS-R1-4	ctACTGGAGTTCAGACGTGTGCTCTTCCGATCT*TATAAT* TTTCCTTAGA AACCACTGCTAACT
HBG-DS-R1-5	ctACTGGAGTTCAGACGTGTGCTCTTCCGATCT*TACAGC* TTTCCTTAG AAACCACTGCTAACT
HBG-DS-R1-6	ctACTGGAGTTCAGACGTGTGCTCTTCCGATCT*TAATCG* TTTCCTTAG AAACCACTGCTAACT
HBG-DS-R1-7	ctACTGGAGTTCAGACGTGTGCTCTTCCGATCT*GACGAC* TTTCCTTAG AAACCACTGCTAACT
HBG-DS-R1-8	ctACTGGAGTTCAGACGTGTGCTCTTCCGATCT*CTCAGA* TTTCCTTAG AAACCACTGCTAACT
HBG-DS-R1-9	ctACTGGAGTTCAGACGTGTGCTCTTCCGATCT*CTATAC* TTTCCTTAGA AACCACTGCTAACT
HBG-DS-R1-10	ctACTGGAGTTCAGACGTGTGCTCTTCCGATCT*CTGGAC* TTTCCTTAG AAACCACTGCTAACT
FGF21-DS-F2	tcgCTCTTTCCCTACACGACGCTCTTCCGATCT*GCGTAG* TCCCCAGCT GAGAAGACACT
FGF21-DS-R1-1	ctACTGGAGTTCAGACGTGTGCTCTTCCGATCT*ATGAGC* ATTGCATCA TCCGTCCAGGC
FGF21-DS-R1-2	ctACTGGAGTTCAGACGTGTGCTCTTCCGATCT*ATTCCT* ATTGCATCAT CCGTCCAGGC
FGF21-DS-R1-3	ctACTGGAGTTCAGACGTGTGCTCTTCCGATCT*CAAAAG* ATTGCATCA TCCGTCCAGGC
FGF21-DS-R1-4	ctACTGGAGTTCAGACGTGTGCTCTTCCGATCT*CAACTA* ATTGCATCA TCCGTCCAGGC
FGF21-DS-R1-5	ctACTGGAGTTCAGACGTGTGCTCTTCCGATCT*CACCGG* ATTGCATCA TCCGTCCAGGC
FGF21-DS-R1-6	ctACTGGAGTTCAGACGTGTGCTCTTCCGATCT*CACGAT* ATTGCATCA TCCGTCCAGGC
FGF21-DS-R1-7	ctACTGGAGTTCAGACGTGTGCTCTTCCGATCT*CACTCA* ATTGCATCA TCCGTCCAGGC
FGF21-DS-R1-8	ctACTGGAGTTCAGACGTGTGCTCTTCCGATCT*CAGGCG* ATTGCATCA TCCGTCCAGGC
FGF21-DS-R1-9	ctACTGGAGTTCAGACGTGTGCTCTTCCGATCT*CATGGC* ATTGCATCA TCCGTCCAGGC
FGF21-DS-R1-10	ctACTGGAGTTCAGACGTGTGCTCTTCCGATCT*CCAACA* ATTGCATCA TCCGTCCAGGC
FGF21-DS-R1-11	ctACTGGAGTTCAGACGTGTGCTCTTCCGATCT*CGGAAT* ATTGCATCA TCCGTCCAGGC
FGF21-DS-R1-12	ctACTGGAGTTCAGACGTGTGCTCTTCCGATCT*CTAGCT* ATTGCATCA TCCGTCCAGGC

The Linkers	*sequence (5'-3')*
Linker-d1	GGATCCAACGGTCCGGGTGGATCTGGAGGTGGAGGTTCTGGAGGA
	AGCCTGGGCAGCGGCTCCCCCAAGAAAAAACGCAAGGTGGAAGATC
	CTAAGAAAAAGCGGAAAGTGGGATCCAACGGTCCGGGTGGATCTGG
Linker-d2	AGGTGGAGGTTCTGGAGGA
	AGCCTGGGCAGCGGCTCCCCCAAGAAAAAACGCAAGGTGGAAGATC
	CTAAGAAAAAGCGGAAAGTGGACGGCATTGGTAGTGGGAGCAACGG
The original linker	CAGCAGCGGATCCAACGGTCCGGGTGGATCTGGAGGTGGAGGTTCT
	GGAGGA

The Nuclear Localization Sequences (NLS)	*sequence (5'-3')*
	AACTCAAAGAAAAGGTCACTGGAGGACAACGAAACGGAAATCAAAGT
	CTCCAGAGACACCTGGAACACTAAGAACATGCGGTCCCTGGAACCGC
Ty1NLS	CACGAAGCAAGAAACGGATACAT
	AAAAGGCCGGCGGCCACGAAAAAGGCCGGCCAGGCAAAAAAGAAAA
NPM NLS	AG
	AAAAGAACCGCCGACGGCAGCGAATTCGAGCCCAAGAAGAAGAGGA
bpNLS	AAGTC
sv40 NLS	CCCAAGAAAAAACGCAAGGTG

tRNA	*sequence (5'-3')*
	TGCACCAGCCGGGAATCGAACCCGGGTCTGTACCGTGGCAGGGTAC
tRNA	TATTCTACCACTAGACCACTGGTGCTTTGTT

VP64	*sequence (5'-3')*
	GATGCTTTAGACGATTTTGACTTAGATATGCTTGGTTCAGACGCGTTA
	GACGACTTCGACCTAGACATGTTAGGCTCAGATGCATTGGACGACTT
	CGATTTAGATATGTTGGGCTCCGATGCCCTAGATGACTTTGATCTAGA
The original VP64	TATGCTA
	GACGCATTGGACGATTTTGATCTGGATATGCTGGGAAGTGACGCCCT
	CGATGATTTTGACCTTGACATGCTTGGTAGTGATGCCCTTGATGACTT
Human codon-	TGACCTCGACATGCTCGGCAGTGACGCCCTTGATGATTTCGACCTGG
optimized VP64	ACATGCTG

Supplementary Methods

Plasmid Construction

1) pHRdSV40-scFv-GCN4-sfGFP-VPR-GB1-NLS

To construct the VPR system, the sequences VP64 (PCR amplified from the Addgene plasmid, # 60904), p65 (PCR amplified from the Addgene plasmid, # 61423) and truncated RTA (125-190 aa, chemically synthesized) separated by a short amino acid linker, were cloned to the plasmid (#60904) to replace VP64 sequence.

2) pRGEN-CMV-dCjCas9

To construct the plasmid d*Cj*Cas9, d*Cj*Cas9 sequence harboring the inactivating D8A H559A was cloned into the plasmid (#89752) to replace *Cj*Cas9 sequence.

3) pRGEN-CMV-p300:dCjCas9 / pRGEN-CMV-dCjCas9:p300 (p300-D/D-p300)

The human p300core amplified from the plasmid pCAG-p300:dLbCpf1[1], was fused with d*Cj*Cas9 sequence at the N- or C-terminus.

4) pRGEN-CMV-SunTag:dCjCas9 / pRGEN-CMV-dCjCas9:SunTag (S-D/D-S)

The 10× GCN4 sequence was amplified from the plasmid (#60910), and fused with d*Cj*Cas9 sequence at the N- or C-terminus.

5) pRGEN-CMV-VPR:dCjCas9 / pRGEN-CMV-dCjCas9:VPR (V-D/D-V)

The VPR sequence was amplified from the plasmid pHRdSV40-scFv-GCN4-sfGFP-VPR-GB1-NLS, and fused with d*Cj*Cas9 sequence at the N- or C-terminus.

6) pRGEN-CMV-VPR:CjCas9 (V-WT)

The VPR sequence was amplified from the plasmid pHRdSV40-scFv-GCN4-sfGFP-VPR-GB1-NLS, and cloned into the plasmid (#89752) at the N- terminus of *Cj*Cas9 sequence.

7) pRGEN-CMV-PR:CjCas9 (PR) / pRGEN-CMV-VR:CjCas9 (VR) / pRGEN-CMV-VP:CjCas9 (VP)

The sequence VP64, p65 or RTA was deleted, respectively. The remaining element PR, VR or VP was cloned into the plasmid V-D to replace the VPR sequence.

8) pRGEN-CMV-VNR:CjCas9 (VNR) / pRGEN-CMV-VRN:CjCas9 (VRN)

The Nanog sequence (232-280 aa) amplified from mouse cDNA was cloned into the plasmid VR at the N- or C-terminus of RTA.

9) pRGEN-CMV-PH:CjCas9 (PH)

The p65 sequence amplified from the plasmid (#61423) was fused with HSF1, and cloned into the plasmid V-D to replace the VPR sequence.

10) pRGEN-CMV-VPR-S:CjCas9 (VPR-S)

VPR with a shortened p65 and shortened linkers between the three transcription factors was cloned into the plasmid V-D to replace the VPR sequence.

11) pRGEN-CMV-VPR:CjCas9-d1 (Linker-d1) / pRGEN-CMV-VPR:CjCas9-d2 (Linker-d2)

To generate the plasmid with a shorter linker to connect VPR and d*Cj*Cas9,

Linker-d1 and Linker-d2 were synthesized and then cloned into plasmid V-D to replace the original linker.

12) pRGEN-CMV-VPR:CjCas9-d1-ΔHNH (ΔHNH) / pRGEN-CMV-VPR:CjCas9-d1-ΔHNH-GSK (ΔHNH GSK) / pRGEN-CMV-VPR:CjCas9-d1-ΔHNH-GS (ΔHNH GS)

To construct the truncated d*Cj*Cas9 system, the HNH domain (495-609 aa) was deleted in the V-D plasmid and the remained N- and C- terminal domains were ligated with no linker, GSK linker or GGGSGG linker.

13) pRGEN-CMV-VPR:CjCas9 codon optization (V-D*****)

DNA encoding the human-codon-optimized VP64 sequence was chemically synthesized and cloned into the V-D plasmid to replace the original VP64 sequence.

14) pRGEN-CMV-VPR-S:CjCas9-d1 (VPR-S-HNH-d1)

The VPR-S sequence and the truncated d*Cj*Cas9 sequence (ΔHNH GSK) were linked by linker-d1 and cloned into the V-D plasmid backbone.

15) pRGEN-CMV-VPR-S:CjCas9-d1 codon optimization (VPR-S-HNH-d1*, namely, miniCAFE)

The VPR-S* sequence harboring human-codon-optimized VP64 sequence and the truncated d*Cj*Cas9 sequence (ΔHNH GSK) were linked by linker-d1 and cloned into the plasmid V-D backbone.

16) pRGEN-CMV-Ty1NLS:VPR:dCjCas9 (Ty1NLS-) / pRGEN-CMV-VPR:Ty1NLS:dCjCas9 (-Ty1NLS-) / pRGEN-CMV-VPR:dCjCas9:Ty1NLS (-

Ty1NLS) / pRGEN-CMV-NPM NLS:VPR:dCjCas9 (NPM NLS-) / pRGEN-CMV-VPR:NPM NLS:dCjCas9 (-NPM NLS-) / pRGEN-CMV-VPR:dCjCas9:NPM NLS (-NPM NLS) / pRGEN-CMV-bpNLS:VPR:dCjCas9 (bpNLS-) / pRGEN-CMV-VPR:bpNLS:dCjCas9 (-bpNLS-) / pRGEN-CMV-VPR:dCjCas9:bpNLS (-bpNLS)

To generate nuclear localization-optimized transcriptional activator constructs, a bipartite Ty1 retrotransposon NLS (Ty1NLS), a bipartite nucleoplasmin NLS (NPM NLS) or a bipartite SV40 NLS (bpNLS) was respectively fused to either the N terminal or C terminal of V-D, or was used to replace the original 2xSV40 NLS between VPR and dCjCas9.

17) pT3-IR-T2A-Puro-pEF1α-V-D-IR / pT3-IR-T2A-Puro-pEF1α- miniCAFE-IR

To generate the donor constructs, the sequence of VPR:dCjCas9 or miniCAFE was amplified from the corresponding plasmids and cloned into the plasmid pT3-IR-T2A-Puro-pEF1α-EGFP-IR[2] to replace EGFP.

18) rAAV-CMV-V-D-WPRE

The sequence of VPR:dCjCas9 was amplified and sub-cloned into AAV backbone construct (supported by Brainvta), which included a CMV promoter, a WPRE element and rAAV components.

19) rAAV-U6-gFgf21-CMV-miniCAFE-WPRE / rAAV-U6-gGFP-CMV-miniCAFE-WPRE

The miniCAFE sequence was amplified and sub-cloned into the plasmid rAAV-CMV-V-D-WPRE to replace VPR:dCjCas9 sequence. The gRNA expression cassette including the human U6 promoter and g*Fgf21* or gEGFP were sub-

cloned into the miniCAFE encoding plasmid.

20) pU6-cj-sgRNA F scaffold (F) / pU6-cj-sgRNA EF scaffold (EF) / pU6-cj-sgRNA 2.1 scaffold (2.1) / pU6-cj-sgRNA 2.2 scaffold (2.2) / pU6-cj-sgRNA GA scaffold (GA)

Various optimized sgRNA scaffolds were synthesized and cloned into the plasmid pU6-cj-sgRNA to replace the original scaffold, and the detailed information about these sgRNA scaffolds was described in supplementary figure 5d.

21) pU6-cj-tRNA-sgRNA

The chemically synthesized tRNA sequences were cloned into the plasmid pU6-cj-sgRNA between the U6 promoter and sgRNAs.

22) p*dpy-30*-miniCAFE

The *dpy-30* promoter (a ubiquitous promoter) sequence was amplified from *C. elegans* N2 strain genomic DNA and cloned into pCMV-miniCAFE plasmid to replace the CMV promoter.

References

1. Zhang, X. et al. Gene activation in human cells using CRISPR/Cpf1-p300 and CRISPR/Cpf1-SunTag systems. *Protein Cell* **9**, 380-383 (2018).
2. Ma, S. et al. Enhancing site-specific DNA integration by a Cas9 nuclease fused with a DNA donor-binding domain. *Nucleic Acids Research* **48**, 10590-10601 (2020).
